# p63 exerts spatio-temporal control of palatal epithelial cell fate to prevent cleft palate

**DOI:** 10.1371/journal.pgen.1006828

**Published:** 2017-06-12

**Authors:** Rose Richardson, Karen Mitchell, Nigel L. Hammond, Maria Rosaria Mollo, Evelyn N. Kouwenhoven, Niki D. Wyatt, Ian J. Donaldson, Leo Zeef, Tim Burgis, Rognvald Blance, Simon J. van Heeringen, Hendrik G. Stunnenberg, Huiqing Zhou, Caterina Missero, Rose Anne Romano, Satrajit Sinha, Michael J. Dixon, Jill Dixon

**Affiliations:** 1 Faculty of Biology, Medicine & Health, Manchester Academic Health Sciences Centre, Michael Smith Building, University of Manchester, Manchester, United Kingdom; 2 CEINGE Biotecnologie Avanzate Scarl (Center for Genetic Engineering), Napoli, Italy; 3 Department of Molecular Developmental Biology, Faculty of Science, Radboud Institute for Molecular Life Sciences, Radboud University, Nijmegen, The Netherlands; 4 Department of Molecular Biology, Faculty of Science, Radboud Institute for Molecular Life Sciences, Radboud University, Nijmegen, The Netherlands; 5 Department of Human Genetics, Radboud University Medical Center, Nijmegen, The Netherlands; 6 Department of Biology, University of Naples, Federico II, Napoli, Italy; 7 Department of Oral Biology, School of Dental Medicine, State University of New York at Buffalo, Buffalo, New York, United States of America; 8 Department of Biochemistry, Jacobs School of Medicine and Biomedical Sciences, State University of New York at Buffalo, Buffalo, New York, United States of America; University of Helsinki, FINLAND

## Abstract

Cleft palate is a common congenital disorder that affects up to 1 in 2500 live births and results in considerable morbidity to affected individuals and their families. The aetiology of cleft palate is complex with both genetic and environmental factors implicated. Mutations in the transcription factor p63 are one of the major individual causes of cleft palate; however, the gene regulatory networks in which p63 functions remain only partially characterized. Our findings demonstrate that p63 functions as an essential regulatory molecule in the spatio-temporal control of palatal epithelial cell fate to ensure appropriate fusion of the palatal shelves. Initially, p63 induces periderm formation and controls its subsequent maintenance to prevent premature adhesion between adhesion-competent, intra-oral epithelia. Subsequently, TGFβ3-induced down-regulation of p63 in the medial edge epithelia of the palatal shelves is a pre-requisite for palatal fusion by facilitating periderm migration from, and reducing the proliferative potential of, the midline epithelial seam thereby preventing cleft palate.

## Author summary

Cleft palate is a serious congenital condition which affects approximately 1 in every 2500 births. Cleft palate occurs when the palatal shelves fail to grow, adhere or fuse during development. Mutations in the gene encoding the transcription factor p63 result in cleft palate in humans and mice. However, the role of p63 and how it controls the network of genes to regulate palate development is not well understood.In this study, we demonstrate that p63 controls the spatio-temporal regulation of palatal epithelial cell fate to ensure appropriate palatal adhesion: p63 induces the formation of a flattened layer of epithelial (periderm) cells and controls its subsequent maintenance. We also demonstrate that TGFβ3-induced, down-regulation of p63 in the medial edge epithelial cells, through which the palatal shelves adhere and fuse, controls *Jag2*-induced periderm migration to the oral and nasal epithelial triangles. In addition, p63 plays a central role in maintaining the proliferative potential of the basal layer of the medial edge epithelia. Our study provides significant new insights into the mechanisms that regulate development of the palate by establishing the role of p63 in governing the fate of the midline epithelial cells.

## Introduction

Cleft palate is a common congenital anomaly with a prevalence estimated at 1:2500 live births, that results from failure of growth, elevation, adhesion and/or fusion of the palatal shelves during embryogenesis [1,2]. Cleft palate causes major morbidity to affected individuals through problems with feeding, breathing, speaking, hearing and social adjustment which can be ameliorated to varying degrees by airway support, surgery, speech therapy, dental treatment, and psychosocial intervention [1,2]. The frequent occurrence and resulting major healthcare burden highlight the need to dissect the mechanisms that underlie development of the secondary palate and how they are disturbed in cleft palate [2].

In mice, development of the secondary palate mirrors that of humans; consequently, the mouse is the main model organism for the study of mammalian palatogenesis [3]. In mice, palatal shelves initiate from the maxillary processes on embryonic day (E)11 and grow vertically, lateral to the tongue, during E12 and E13. At these stages, each palatal shelf consists of a core of neural crest cell-derived mesenchyme surrounded by a simple, undifferentiated epithelium consisting of a basal layer of cuboidal ectodermal cells and a surface layer of flattened periderm cells [4,5]. During E14, the palatal shelves re-orientate to a horizontal position above the tongue and contact via their medial edge epithelia (MEE). The MEE of the apposed palatal shelves adhere to form a midline epithelial seam (MES) which subsequently degenerates to allow mesenchymal continuity across the palate by E15.5 [3].

Although the epithelia of the vertical palatal shelves are in intimate contact with the mandibular and lingual epithelia, pathological fusions between the palate and the mandible and/or the tongue are rare [6–8]. Nevertheless, the MEE must rapidly acquire the capability to fuse if the palatal shelves are not to remain cleft. Although the mechanisms by which MES degeneration is completed have been controversial, the prevailing evidence supports a major role for cell death [9–11]. Moreover, competence for palatal shelf fusion is precisely regulated: periderm acts as a barrier which prevents premature adhesion of the oral epithelia and removal of periderm from the MES is therefore a prerequisite for palatal fusion [8,12]. However, the molecular mechanisms underlying the precise spatio-temporal control of palatal adhesion/fusion remain incompletely characterized.

Mutations in the gene encoding the transcription factor p63 result in cleft palate in humans and mice [13,14]. The *TP63* gene encodes at least six protein variants. Different promoters produce two alternative N-termini; TA-isoforms which contain a transactivation sequence and ΔN-isoforms which possess a shorter activation domain [13]. In addition, both TA and ΔN isoforms undergo alternative splicing towards the C-terminus giving rise to α-, β- and γ-isoforms [13]. All isoforms contain a DNA-binding domain but they vary in their ability to activate or repress their target genes [15–17]. ΔNp63α, the major isoform present in the oral ectoderm [18], is expressed in the basal epithelial cells until after palatal shelf elevation when it is down-regulated in the midline epithelial seam [19]. Despite the demonstration that p63 transcriptionally activates *Fgfr2* to control epithelial and mesenchymal proliferation during palatal growth [20], cell adhesion molecules to control ectodermal adhesion [21,22], and *Irf6* to regulate palatal fusion [19], it is unclear whether down-regulation of p63 in the MEE is a pre-requisite for, or a consequence of, palatal fusion.

In the current paper, we use the well-characterized *Tgfb3*^-/-^ mutant mouse, which exhibits cleft palate [23], to demonstrate that down-regulation of p63 in the MEE is essential for palatal fusion and that over-expression of ΔNp63α *in vivo* results in sub-mucous cleft palate. In addition, we show that ΔNp63α plays a central role in the spatio-temporal regulation of palatal epithelial cell fate to ensure appropriate adhesion and fusion of the palatal shelves, thereby preventing cleft palate.

## Results

### Rescue of cleft palate in *Tgfb3*^-/-^ mice by reducing p63 dosage in the medial edge epithelia

**Fig 1:**
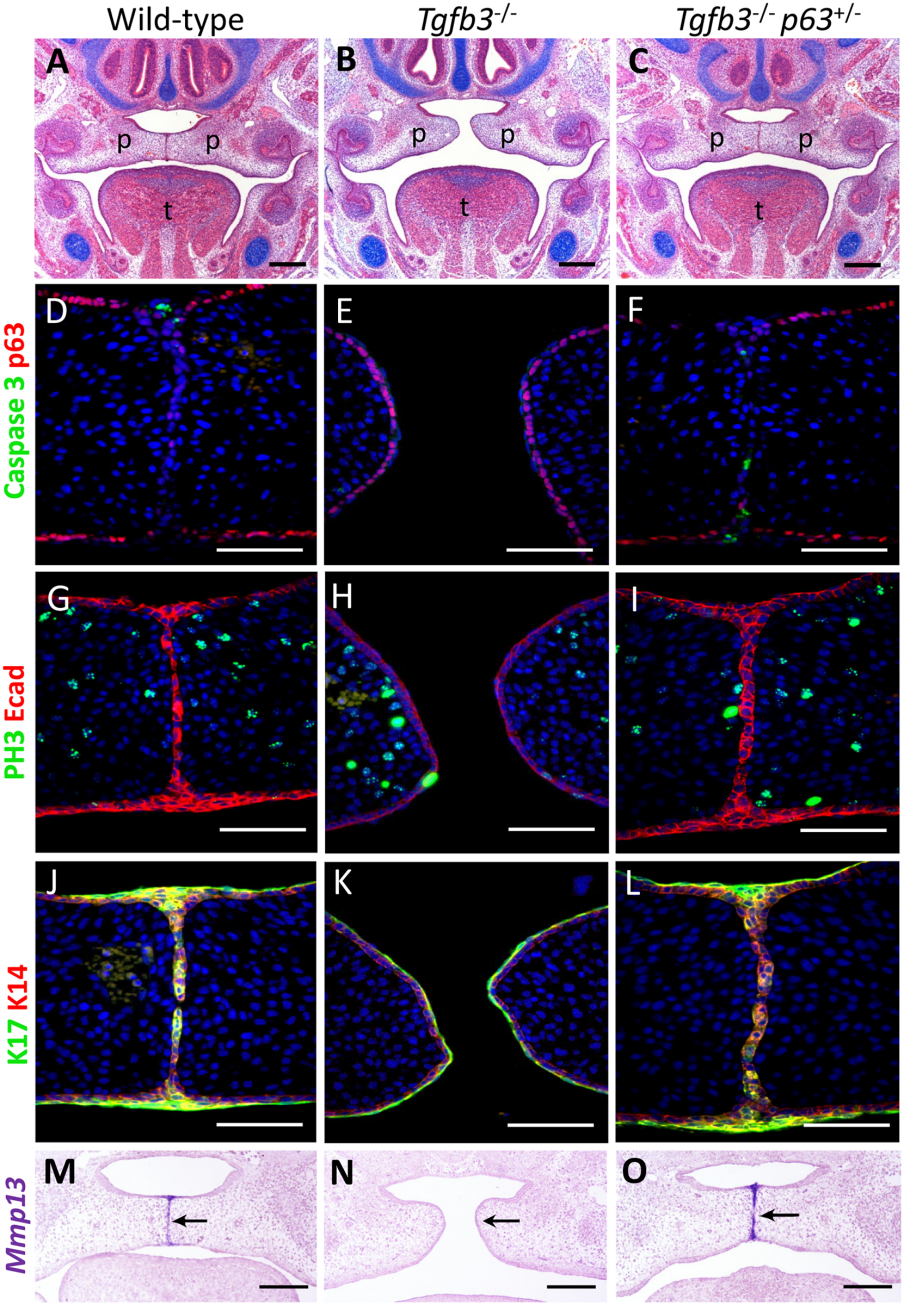
Rescue of cleft palate in *Tgfb3*^-/-^ mice by reducing p63 dosage in the medial edge epithelia. (**A**) The palatal shelves of wild-type mice elevate, adhere and fuse to form a transient midline epithelia seam at E14.5 while (**B**) the palatal shelves of *Tgfb3*^-/-^ embryos elevate but fail to adhere to, or fuse with, one another. (**C**) In contrast, reducing p63 dosage in a *Tgfb3*^-/-^ background restores the wild-type phenotype. (**D—I**) In E14.5 wild-type mice, p63 expression is down-regulated in the MEE cells which cease proliferation and undergo apoptosis. In contrast, in E14.5 *Tgfb3*^-/-^ mice, p63 expression is maintained in the MEE which continue to proliferate and do not undergo cell death. Reducing p63 dosage in a *Tgfb3*^-/-^ background restores wild-type behaviour in the MEE. (**J—L**) In wild-type and *Tgfb3*^-/-^;*p63*^+/-^ mice, keratin 17-positive periderm cells migrate out of the MEE to form the oral and nasal epithelial triangles, whereas in *Tgfb3*^-/-^ mice, distinct keratin 14-positive basal and keratin 17-positive periderm layers persist and the palatal shelves fail to adhere/fuse. (**M—O**) *Mmp13*, which is absent from the MEE in E14.5 *Tgfb3*^-/-^ mice (arrowed), is restored to a wild-type expression pattern in *Tgfb3*^-/-^;*p63*^+/-^ embryos. p: palatal shelves; t: tongue. Scale bars: A-C & M-O, 250 μm; D-L, 100 μm.

As p63 expression is maintained in the MEE of *Tgfb3*^-/-^ mice which exhibit cleft palate and persistent periderm cells over the MEE [12,19,23,24], we manipulated the level of p63 in the MEE of *Tgfb3*^-/-^ mice genetically. Initially, we generated *Tgfb3*^+/-^;*p63*^+/-^ compound heterozygous mice which did not exhibit any gross abnormalities and in which fusion of the secondary palate proceeded normally. These mice were inter-crossed with *Tgfb3*^+/-^ mice to generate *Tgfb3*^-/-^ embryos which were also heterozygous for *p63*. Histological analysis demonstrated that up to E13.5 palatal development progressed normally in wild-type, *Tgfb3*^-/-^, and *Tgfb3*^-/-^;*p63*^+/-^ mice (S1 Figure). In contrast, while the vast majority of E14.5 (Fig 1A–1C) and E15.0 (S2 Figure) *Tgfb3*^-/-^;*p63*^+/+^ mice exhibited cleft palate, consistent with previous reports [23], the MEE of the *Tgfb3*^-/-^;*p63*^+/-^ littermates approximated and adhered in the midline to form the MES which subsequently degenerated to allow mesenchymal continuity across the palate in 88.9% of embryos (n = 24/27). Although 6.9% (n = 2/29) of *Tgfb3*^-/-^embryos displayed a small degree of palatal shelf fusion which was restricted to the anterior region of the secondary palate, this difference is statistically significant (p<0.001; Fisher’s Exact Test) and supports the hypothesis that epistatic down-regulation of p63 in *Tgfb3*^-/-^ embryos rescues the cleft palate phenotype.

### Down-regulation of p63 in *Tgfb3*^-/-^ mice restores medial edge epithelial fate

Previous research has shown that cessation of proliferation, induction of apoptosis, and periderm removal is essential for completion of palatal fusion [11,25], we therefore characterized MEE fate in wild-type, *Tgfb3*^-/-^ and *Tgfb3*^-/-^;*p63*^+/-^ mice in greater detail. At E13.5, palatogenesis in *Tgfb3*^-/-^ and *Tgfb3*^-/-^;*p63*^+/-^ mice was comparable to that observed in their wild-type littermates (S1 Figure). At E14.5, p63 expression persisted in the basal cells of the epithelia of the cleft palatal shelves in *Tgfb3*^-/-^ mice; in contrast, the MES of wild-type and *Tgfb3*^-/-^;*p63*^+/-^ embryos had formed, with no apparent p63 staining observed in the midline seam (Fig 1D–1F). Phosphohistone-H3 immunostaining demonstrated that the MEE ceased proliferation in *Tgfb3*^-/-^;*p63*^+/-^ embryos, with no significant difference in proliferation in the palatal epithelium or mesenchyme between wild-type or *Tgfb3*^-/-^;*p63*^+/-^ embryos (S3 Figure) while activated caspase-3 immunostaining confirmed apoptotic activity in the MEE of *Tgfb3*^-/-^;*p63*^+/-^ mice, thereby restoring the pattern of cell behaviour exhibited by wild-type mice (Fig 1D–1I). In addition, keratin 14 and keratin 17 immunostaining indicated that the distinct basal and periderm cell layers that characterize the MEE of *Tgfb3*^-/-^ mice, were restored to a wild-type pattern in *Tgfb3*^-/-^;*p63*^+/-^ embryos (Fig 1J–1L). Similarly, *in situ* hybridization for *Mmp13*, which is expressed in the MEE of wild-type embryos but lost in their *Tgfb3*^-/-^ littermates, was restored in *Tgfb3*^-/-^;*p63*^+/-^ mice (Fig 1M–1O).

**Fig 2:**
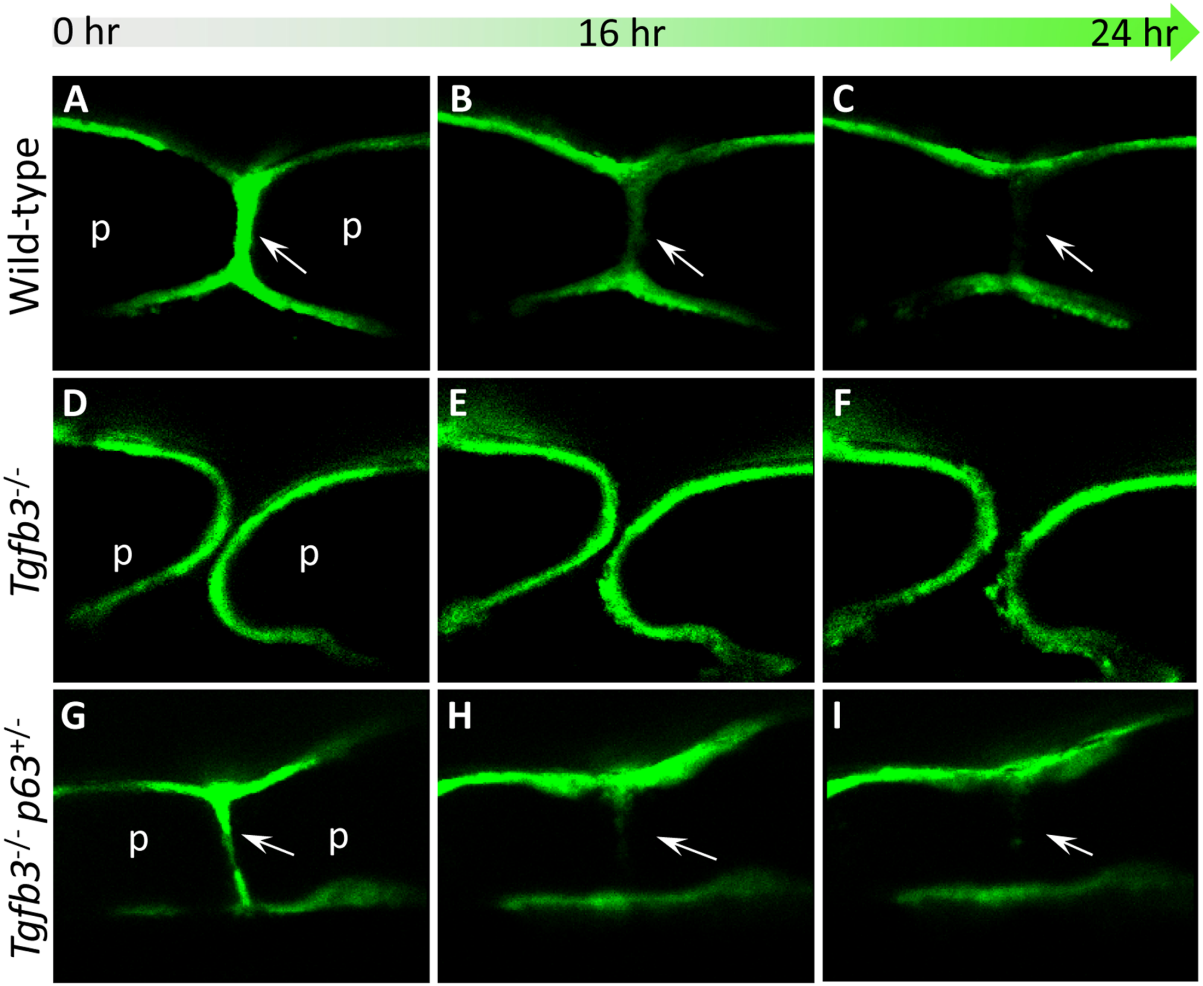
Down-regulation of p63 in the medial edge epithelia allows periderm migration. *mKrt17*-GFP transgenic mice were used for time-lapse confocal imaging of periderm migration during development of the secondary palate. (**A-C**) On a wild-type background, GFP-positive periderm cells migrate out of the midline seam (arrowed) to form the epithelial triangles on the oral and nasal surfaces as part of the process whereby mesenchymal continuity across the palate is achieved. (**D-F**) In contrast, in *Tgfb3*^-/-^ embryos, GFP-positive periderm cells fail to migrate out of the midline epithelial seam and the secondary palate remains cleft. (**G-I**) Reducing p63 dosage in *Tgfb3*^-/-^ embryos restores the migratory periderm phenotype allowing palatal fusion and rescuing the cleft palate phenotype (region of midline is arrowed). p: palatal shelves. The images are representative stills taken at the same Z position over a 24-hour culture period. The videos are provided as Supplemental Material, S1–S3 Videos.

To study periderm fate in greater detail, a transgenic reporter mouse in which the 5’ upstream sequence from the mouse *keratin 17* gene directs GFP expression in ectoderm-derived epithelial appendages during embryonic development (*mKrt17*-GFP) [26], was employed in combination with an *in vitro* palatal shelf culture system. During palatogenesis in wild-type embryos, confocal imaging of GFP expression over a 24-hour period revealed that periderm cells migrated out of the MEE towards the epithelial triangles and into the oral and nasal epithelia of the palatal shelves allowing MES degradation to be completed (S1 Video). As p63 is down-regulated in the MEE of wild-type but not *Tgfb3*^-/-^ mice which exhibit cleft palate and persistent periderm cells over the MEE [12,19], we crossed the *mKrt17*-GFP reporter onto a *Tgfb3*^-/-^ background and noted that GFP-labelled periderm cells failed to migrate out of the MEE despite forced contact between the palatal shelves (Fig 2D–2F; S2 Video). In contrast, the migratory periderm phenotype observed in the MEE of wild-type mice was restored in *Tgfb3*^-/-^;*p63*^+/-^ embryos (Fig 2G–2I; S3 Video).

### p63 regulates a cell adhesion network in the secondary palate

**Fig 3:**
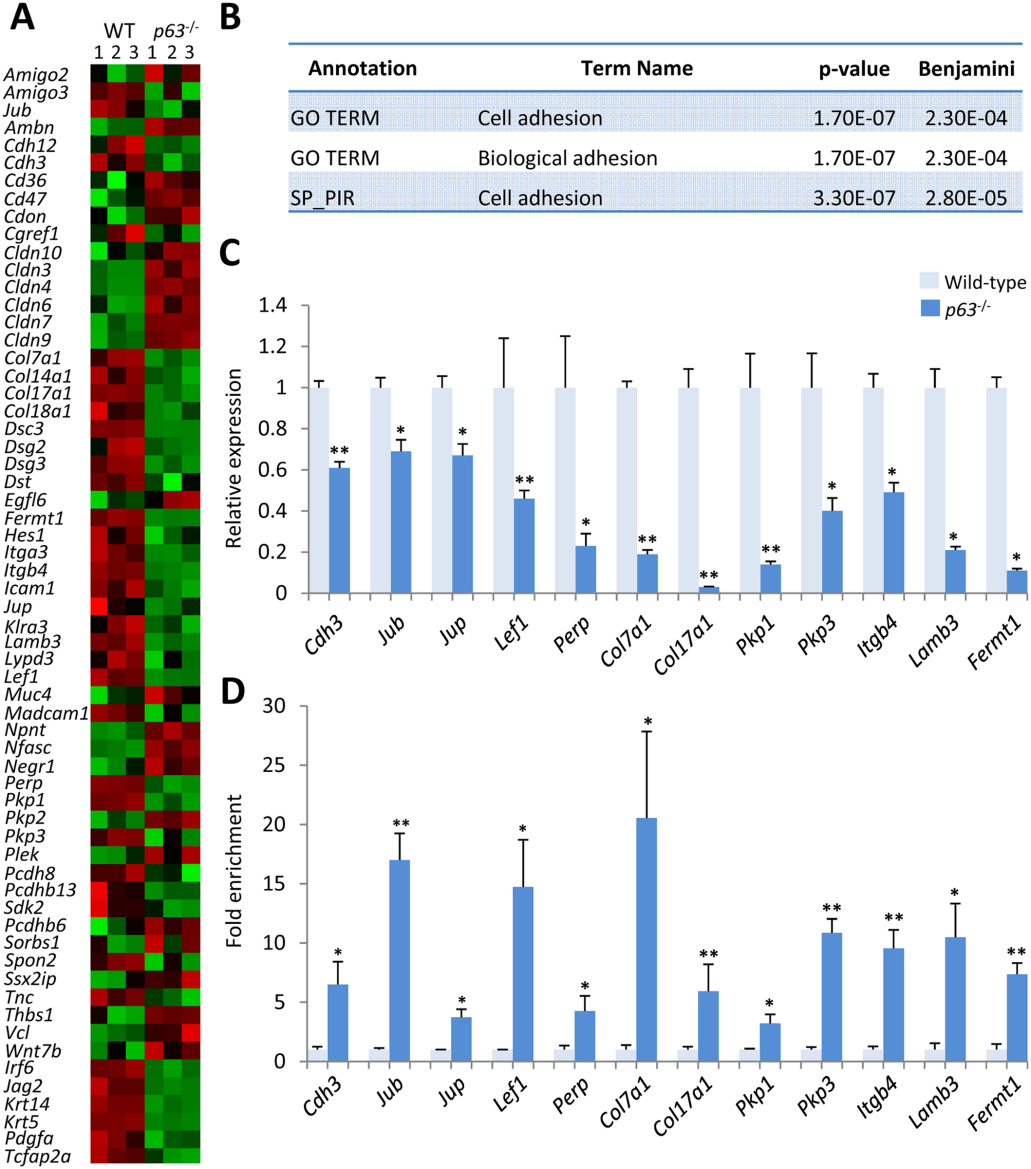
p63 regulates an adhesion programme in the secondary palate. (**A**) Heatmap of genes encoding proteins involved in cell adhesion that are differentially-expressed in the palatal shelves of wild-type *versus p63*^-/-^ embryos (green, down-regulation; black, no significant change; red, up-regulation). (**B**) Functional annotation of differentially-expressed genes in the palatal shelves of wild-type *versus p63*^-/-^ embryos as assessed by DAVID analysis. (**C**) qPCR analysis of genes indicated as differentially-regulated in microarray analyses using RNA extracted from palatal shelves dissected from E14.0 wild-type and *p63*^-/-^ mice. (**D**) ChIP-qPCR analysis of p63-bound sites within 25 kb of cell adhesion genes. Fold-enrichment for each binding region was calculated relative to a control region in exon 2 of myoglobin (set at 1; pale bar), to which p63 does not bind. Asterisks represent level of significance: * = P <0.05, ** = P <0.01; Student’s t-test (C), n = 4; Mann Whitney U test (D), n = 5 for each genotype.

To gain insights into the molecular networks controlled by p63 during development of the secondary palate, we performed chromatin immunoprecipitation followed by high-throughput sequencing (ChIP-seq) on pooled E13.5/E14.5 mouse palatal shelves and identified 6295 genomic regions bound by ΔNp63α *in vivo* (p ≤1e-5) (S4 Figure, S1 Table). Gene-set enrichment performed using the Genomic Regions Enrichment of Annotation Tool (GREAT) [27] indicated that p63 binding sites were significantly enriched close to genes encoding proteins implicated in ectodermal development including cell junction organization/assembly, adherens junctions, and hemi-desmosome assembly suggesting that ΔNp63α plays an important role in controlling adhesion of the palatal shelves during development (S4 Figure). Subsequently, we integrated the ChIP-seq data with microarray data obtained from palatal shelves dissected from individual E14 wild-type *versus p63*^-/-^ embryos and identified 889 differentially-expressed genes (p<0.05: 441 genes down-regulated; 448 up-regulated) (Fig 3A; S2 Table). Consistent with the GREAT analysis, Gene Ontology analysis indicated that genes involved in ectodermal development and cell adhesion were over-represented among the differentially-expressed genes (Fig 3B; S3 Table). Down-regulated genes included those involved in desmosome formation, adherens junction formation, and cell-matrix adhesion. Conversely, up-regulated genes included those involved with tight junction formation (Fig 3; S3 Table). RT-qPCR analysis confirmed the results of the microarray analysis (Fig 3C) while ChIP-qPCR validated p63 binding in close proximity to these genes *in vivo* (Fig 3D) strongly suggesting that ΔNp63α transcriptionally regulates a cell adhesion network in the secondary palate.

### p63 function is essential for periderm development

**Fig 4:**
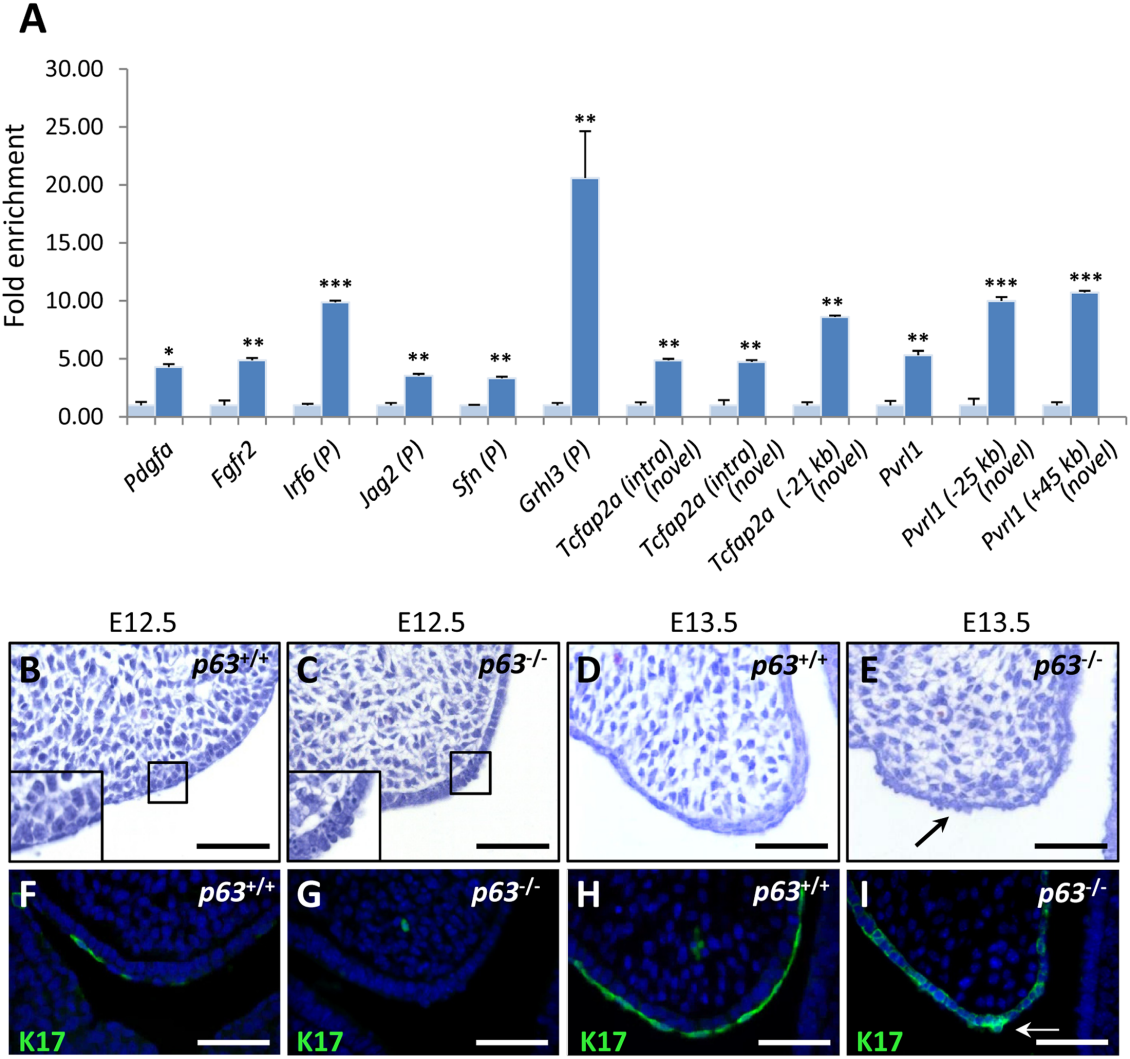
p63 is essential for periderm development. (**A**) ChIP-qPCR validation of p63-bound sites within regulatory regions surrounding ‘cleft palate-associated genes’. Fold-enrichment for each binding region was calculated relative to a control region in exon 2 of myoglobin (set at 1; pale bar), to which p63 does not bind. (P) Genes associated with periderm formation; Intra, intragenic. Asterisks represent level of significance: * = P <0.05, ** = P <0.01, *** = P <0.001. Student’s t-test, n = 4. (**B**-**E**) Histological analysis of palatal epithelia at E12.5 and E13.5. (**B** and **D**) Coronal sections of wild-type palatal shelves at E12.5 and E13.5 reveal a layer of flattened periderm cells overlying the basal epithelia. (**C** and **E**) In *p63*^-/-^ palatal shelves, the flattened cells are absent and individual ‘bead-like’ cells are observed above the basal epithelia at E12.5 (**C**) and E13.5 (**E**). Boxed insets represent higher magnification of the respective regions. (**F**-**I**) Keratin 17 (K17) expression at E12.5 and E13.5. (**F**) K17 is expressed in a number of flattened cells overlying the basal epithelia in wild-type palatal shelves at E12.5. (**G**) In contrast, K17 is not detected in *p63*^-/-^ palatal epithelia at E12.5. (**H**) From E13.5, K17 expression is observed throughout the periderm layer in wild-type palatal epithelia. (**I**) However, K17 expression is absent in *p63*^-/-^ palatal shelves although ectopic K17 expression is detected in basal epithelial cells at E13.5 (arrow). Scale bars: 50 μm.

The ChIP-seq data also identified p63-bound regions associated with *Pvrl1*, *Irf6*, *Fgfr2*, *Tcfap2a*, *Pdgfa*, *Sfn*, *Grhl3* and *Jag2*, mutations in which result in cleft palate [8, 28–31]. With the exception of *Fgfr2* and *Grhl3*, these genes were down-regulated in *p63*^-/-^ palatal shelves. In total, 12 p63 binding sites in ‘cleft palate-associated genes’, including novel sites close to *Pvrl1*, *Tcfap2a*, *Sfn*, *Grhl3* and *Jag2*, were validated by ChIP-qPCR analysis of E13.5 palatal shelves (Fig 4A). Notably, *Irf6*, *Sfn*, *Grhl3* and *Jag2* play an essential role in periderm formation [8,31]. To determine if p63 is required for periderm development, we examined the palatal shelves of E12.5 and E13.5 *p63*^-/-^ mice. Histological analysis of wild-type palatal shelves revealed basal epithelial cells covered by a layer of flattened cells, a morphological characteristic of periderm (Fig 4B and 4D). In contrast, the palatal shelves of *p63*^-/-^ mice lacked this layer with only protruding, rounded cells observed above a disorganized basal cell layer (Fig 4C and 4E). To confirm these results, we analysed keratin 17 expression [32]. While keratin 17-positive periderm cells were observed in wild-type palatal shelves (Fig 4F and 4H), only patchy keratin 17 expression was observed in the basal epithelia of their *p63*^-/-^ littermates (Fig 4G and 4I).

**Fig 5:**
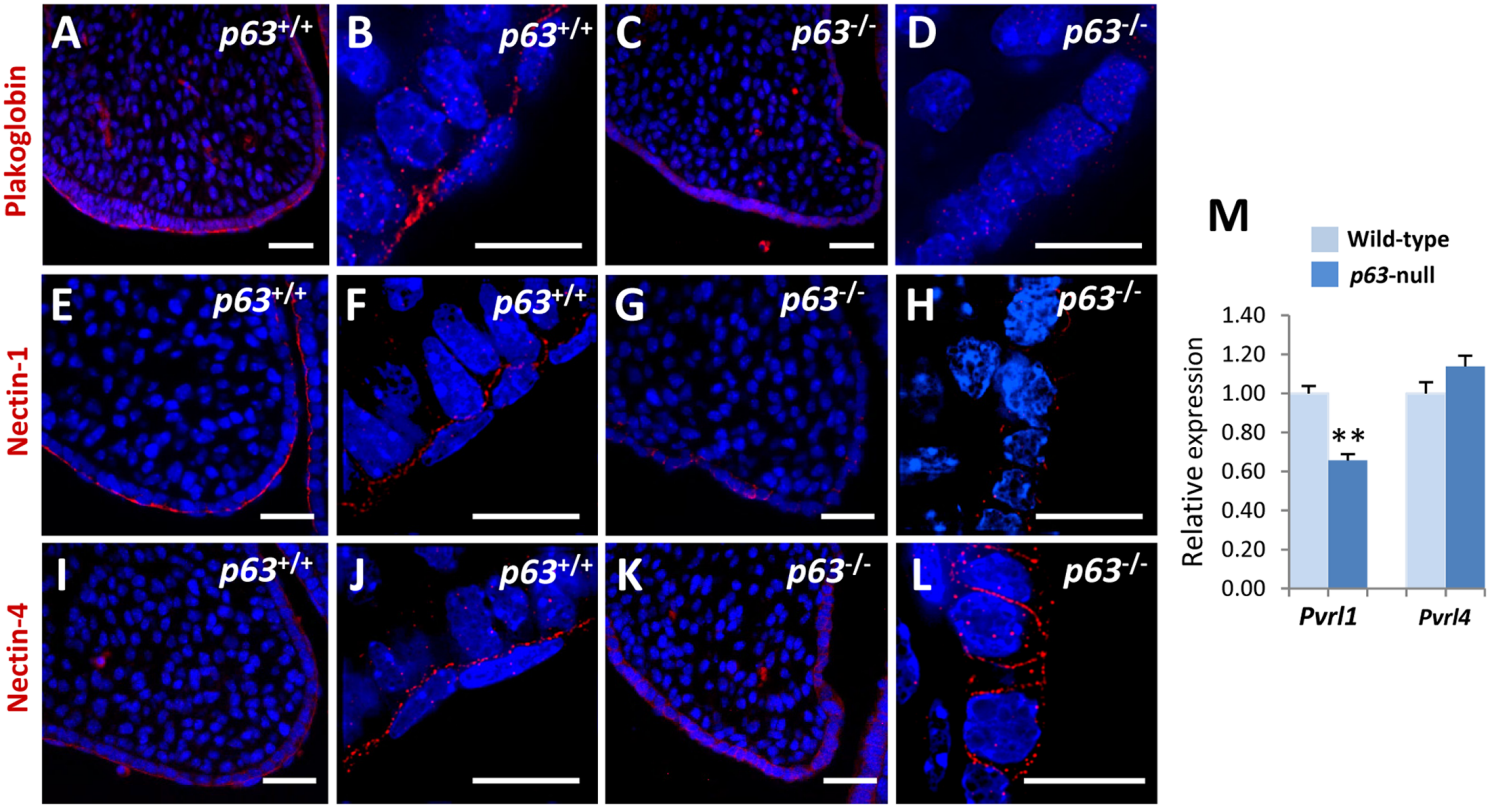
Loss of p63 results in adhesion defects in palatal epithelia. Immunofluorescence analysis of (**A**, **B**) plakoglobin, (**E**, **F**) nectin-1, and (**I**, **J**) nectin-4 reveals strong expression of these proteins at the junction between the periderm/basal cells in the palatal epithelia of E13.5 wild-type mice. (**C**, **D**, **G** and **H**) In contrast, plakoglobin and nectin-1 expression are markedly down-regulated in the E13.5 *p63*^-/-^ palatal epithelia. (**K** and **L**) Nectin-4 expression levels in *p63*^-/-^ palatal shelves are comparable to those of wild-type mice; however deconvolution images reveal that expression of nectin-4, which is normally restricted to the periderm/basal junction, is mis-localized in the epithelia of E13.5 *p63*^-/-^ palatal shelves and is expressed between adjacent basal cells. (**M**) qPCR analysis of palatal shelves dissected from E14.0 wild-type and *p63*^-/-^ mice indicates that *Pvrl1* transcripts are significantly reduced in *p63*^-/-^ palatal shelves while *Pvrl4* levels are comparable to wild-type.** = P <0.01, Mann Whitney U test, n = 5 for each genotype. Scale bars: A, C, E, G, I, K, 50 μm; B, D, F, H, J, L, 20 μm.

To investigate whether p63 regulates adhesion between periderm and basal epithelial cells, we analysed the expression of a subset of cell adhesion molecules identified as p63 targets in the secondary palate. Expression of the desmosomal protein plakoglobin was detected on the surface of the basal epithelial layer of E13.5 wild-type palatal shelves (Fig 5A and 5B). In contrast, plakoglobin expression was markedly reduced in E13.5 *p63*^-/-^ palatal shelves and expression at the apical border of the basal cells was absent (Fig 5C and 5D). Similar to plakoglobin, nectin-1 expression was observed along the basal epithelial layer in E13.5 wild-type palatal shelves (Fig 5E), localizing specifically to the basal/periderm junction (Fig 5F). In contrast, nectin-1 expression was markedly down-regulated in E13.5 *p63*^-/-^ palatal shelves (Fig 5G). Deconvolution images revealed that the localized expression of nectin-1 at the basal/periderm junction was absent in E13.5 *p63*^-/-^ palatal shelves (Fig 5H).

To determine if loss of expression of adherens junction proteins was specific to p63 transcriptional targets, we analysed nectin-4 expression. Mutations in *PVRL4*, which encodes nectin-4, have been found in ectodermal dysplasia syndromes [33], but *Pvrl4* is not thought to be under the control of p63 [22]. In wild-type embryos at E13.5, nectin-4 was localized at the basal/periderm junction throughout the palatal epithelia in a pattern similar to that of nectin-1 (Fig 5I and 5J). In contrast, in the palatal epithelia of E13.5 *p63*^-/-^ mice, nectin-4 expression was mis-localized and was detected ectopically between adjacent basal cells (Fig 5K and 5L). As the microarray expression data had indicated that *Pvrl4* transcript levels were similar in the E13.5 palatal shelves of wild-type and *p63*^-/-^ embryos (S2 Table), we confirmed these findings by qPCR analysis which revealed that total transcript levels of *Pvrl4* were unaltered despite the mis-localization of nectin-4 (Fig 5M). Taken together, these results support the hypothesis that down-regulation of ΔNp63α in the MEE is essential to ensure periderm migration from the MES through its regulation of the ectodermal adhesion programme.

### Over-expression of ΔNp63α in the medial edge epithelia causes sub-mucous cleft palate

To examine further the effect of manipulating the levels of p63 in the MEE, we expressed ΔNp63α ectopically in the palatal epithelia using a transgenic approach. Here, we inter-crossed transgenic mice in which HA-tagged ΔNp63α is under the control of a tetracycline-inducible response element with *Krt5*-tTA transgenic mice in which the tetracycline-regulated, transcriptional transactivator is suppressed by doxycycline to generate *Krt5*-tTA;pTRE-ΔNp63α bi-transgenic mice [34–36]. Histological analysis of secondary palate development in E13.5 *Krt5*-tTA;pTRE-ΔNp63α bi-transgenic mice demonstrated that they exhibited similar palatal morphology to their wild-type littermates, although thickening of the basal epithelial layer was observed in the region of the future MEE and on the oral surface of the palate (S5 Figure; n = 11). Immunostaining with a panel of antibodies confirmed that the periderm developed appropriately and all other aspects of cell behaviour appeared comparable to wild-type littermates (S5 Figure). Phosphohistone H3 staining revealed no significant difference in the percentage of cells proliferating in the epithelium or the mesenchyme in the bi-transgenic mice compared to wild-type littermates (S6 Figure).

**Fig 6:**
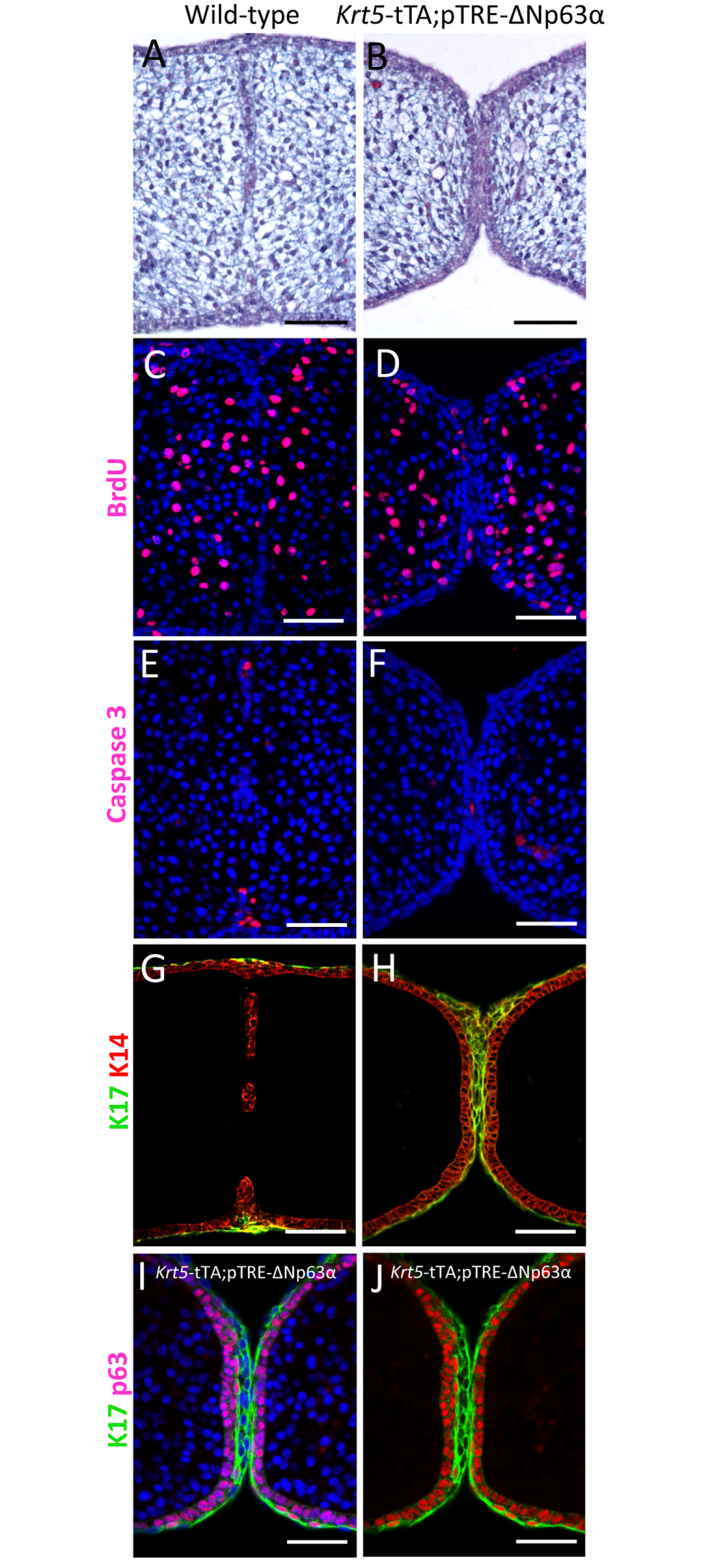
Development of the palatal shelves in E14.5 *Krt5*-tTA;pTRE-ΔNp63α bi-transgenic mice. (**A**) The horizontal palatal shelves of wild-type embryos have formed a midline epithelial seam which has started to degenerate. (**B**) In contrast, the palatal shelves of *Krt5*-tTA;pTRE-ΔNp63α embryos have approximated and adhered via a thickened epithelial midline. (**C,D**). BrdU immunostaining confirmed that the persistent MES continued to proliferate with no evidence of apoptosis (**E, F**). (**G-J**) Immunostaining revealed a persistent double layer of keratin 17-positive periderm cells and an underlying layer of p63-positive basal cells. Scale bars: 50 μm.

Examination of eight litters of embryos at E14.5 or older, when all wild-type littermates exhibited a fully fused and degenerating midline epithelial seam, indicated that 70% (n = 14) of *Krt5*-tTA;pTRE-ΔNp63α bi-transgenic embryos displayed a persistent midline epithelial seam throughout the entire anterior-posterior axis of the palate which varied in thickness, sometimes appearing as ‘rounded-off’ palatal shelves (S7 Figure and Fig 6). The remaining embryos (n = 6) exhibited a combination of phenotypes, including open palatal shelves (n = 3), one shelf elevated/one shelf vertical (n = 2) and one exhibited oral fusions that prevented the palatal shelves from elevating. One litter was discovered on post-natal day 0 with two *Krt5*-tTA;pTRE-ΔNp63α bi-transgenic neonates. Histological sections of these embryos revealed that the palate appeared thin and the shelves were joined by an epithelial ‘bridge’ with no mesenchymal continuity across the palate (S8 Figure).

Immunofluorescence analysis of the E14.5 bi-transgenic embryos which exhibited a persistent MES revealed that occasional proliferative cells were present in the epithelium, unlike wild-type littermates in which the seam cells had ceased proliferation (Fig 6). However, anti-BrdU staining revealed no significant difference in the percentage of cells proliferating either in the epithelium or the mesenchyme between wild-type or bi-transgenic littermates (S9 Figure). Activated caspase 3 staining revealed occasional dying cells in the oral and nasal epithelial triangles in the wild-type embryo compared to very infrequent cells in the *Krt5*-tTA;pTRE-ΔNp63α bi-transgenic embryos, although this was not quantitated (Fig 6E and 6F). Immunofluorescence analysis using K17 indicated that the midline seam in the *Krt5*-tTA;pTRE-ΔNp63α bi-transgenic embryos contained a thick layer of periderm cells with anti-p63 immunostaining demonstrating the proliferative nature of the basal layer (Fig 6G–6J). Immunofluorescence analysis of E15.5 embryos with anti-HA and anti-p63 antibodies confirmed transgene expression in the MEE thereby driving ectopic ΔNp63α expression in these cells (S8 Figure).

### Molecular consequences of increasing p63 expression in the medial edge epithelia

Based on the above results, we performed microarray analysis of palatal shelves dissected from E14.5 bi-transgenic mice and their wild-type littermates. Principal Components Analysis indicated that the results fell into two broad groups, one of which segregated with those obtained from analysis of wild-type mice. Analysis of the results that segregated away from wild-type mouse data led to the identification of 3125 upregulated genes (p<0.05) including numerous p63 transcriptional targets; for example: *Krt5*; *Col7a1*; *Trp73*; *Pkp1*; *Pkp3*; *Ripk4*; *Perp*, *Lamb3* (S4 Table).

Given the large number of differentially-expressed genes, we intersected the results with those obtained from microarray analysis of E14.5 *p63*^-/-^ palatal shelves and selected genes that exhibited a diametric expression pattern. One hundred and four genes identified as being down-regulated in the *p63*^-/-^ microarray were up-regulated in the *Krt5*-tTA;pTRE-ΔNp63α microarray (S5 Table). Enrichment analysis (http://amp.pharm.mssm.edu/Enrichr/) of these genes indicated that the most significantly enriched Gene Ontology terms identified were ‘epithelium’ and ‘epidermis development’. Clustergram analysis, ranked on P value, highlighted several genes known to be important in periderm and craniofacial development, including the known p63 targets *Jag2* [6], *Znf750* [37] and *Perp* [38], as well as *Bcl11b* which has not been implicated in p63 signalling previously. In the latter case, we identified p63 binding to an active enhancer approximately 48 kb upstream of the transcription start site of *Bcl11b*.

**Fig 7:**
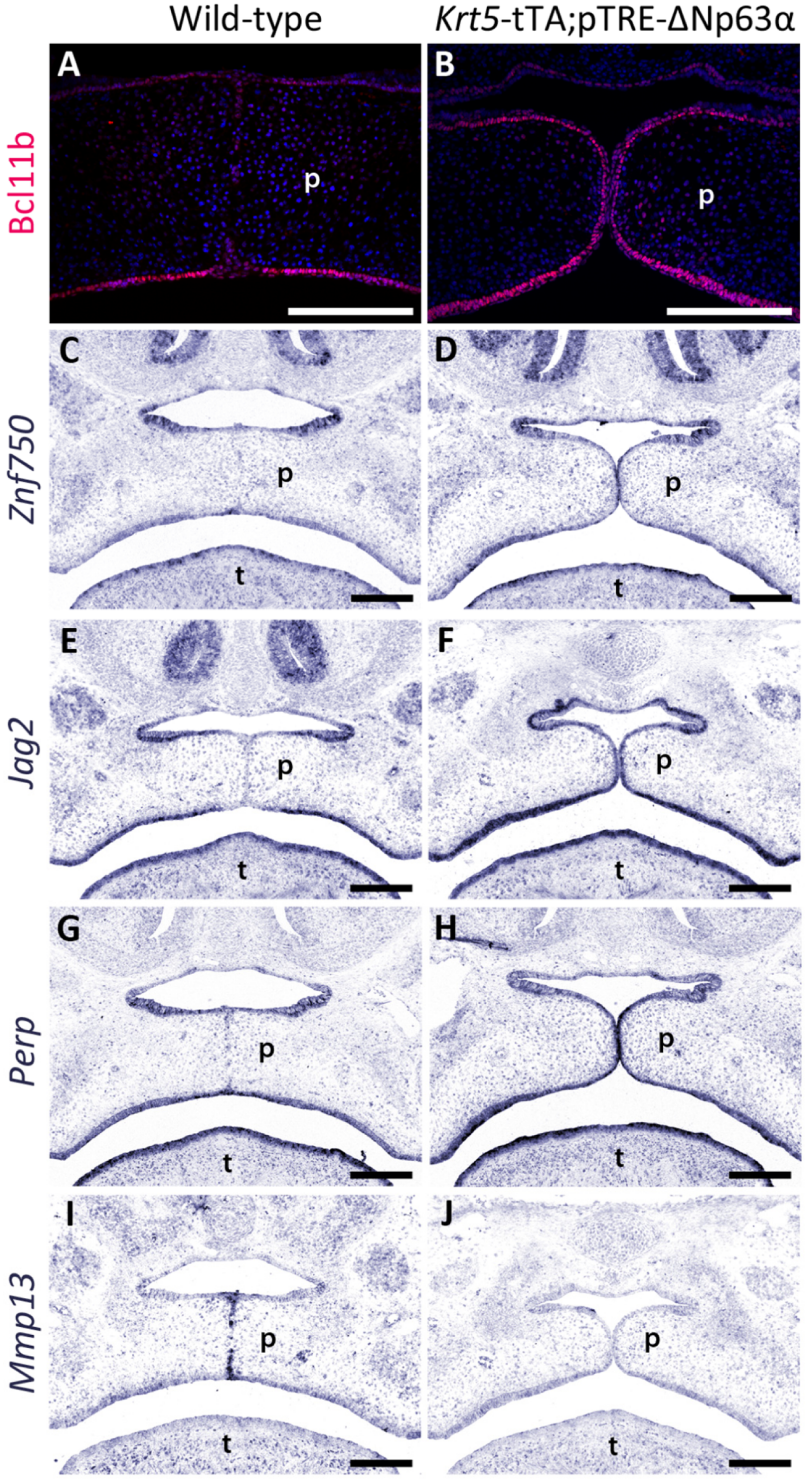
Molecular consequences of increasing p63 expression during palatal development. (**A, B**) The expression of Bcl11b (**A, B**), *Znf750* (**C, D**), *Jag2* (**E,F**) and *Perp* (**G, H**), all of which are down-regulated in the medial edge epithelia of wild-type mice, is maintained in the medial edge epithelia of their *Krt5*-tTA;pTRE-ΔNp63α bi-transgenic littermates. In contrast, *Mmp13* which is normally expressed in the midline epithelial seam of wild-type mice as they adhere until degeneration of the seam, is down-regulated in the medial edge epithelia of *Krt5*-tTA;pTRE-ΔNp63α embryos. p: palatal shelves; t: tongue. Scale bars: 200 μm.

A combination of *in situ* hybridization and immunohistochemistry indicated that while Bcl11b, *Znf750*, *Jag2* and *Perp* were down-regulated in the MEE of wild-type mice immediately prior to adhesion/fusion of the palatal shelves, their expression was maintained in the abnormal MEE of *Krt5*-tTA;pTRE-ΔNp63α mice (Fig 7A–7H). In contrast, *Mmp13* which is present in the midline epithelial seam of wild-type mice was not expressed in ~50% of *Krt5*-tTA;pTRE-ΔNp63α embryos (Fig 7I and 7J).

## Discussion

Although previous research has identified a large number of genes that are transcriptionally regulated by p63 to control epithelial development and differentiation, these studies have been performed *in vitro* using a variety of cell lines including differentiating and terminally-differentiated keratinocytes [37,39–42]. The lack of genome-wide studies of p63 binding performed *in vivo* has therefore limited our ability to dissect the molecular events controlled by p63 in specific developmental systems such as the secondary palate. By performing functional annotation analysis of microarray data generated from the palatal shelves of *p63*^-/-^ embryos and intersecting them with the ChIP-seq data, we have demonstrated that p63 orchestrates a cell adhesion network in the palatal epithelia, positively regulating formation of adherens junction and desmosome complexes while suppressing tight junction formation in palatal epithelia. These findings highlight the importance of p63 in regulating the balance of normal adhesion junction formation in palatal epithelia. These findings are in agreement with previous studies which have shown that p63 plays a critical role in transcriptionally regulating adhesion molecules in keratinocytes and mammary epithelial cells [40].

McDade and co-workers noted that several p63 transcriptional targets identified from human keratinocytes are associated with cleft palate [42]. Consistent with this work, we identified a large number of p63 binding sites that are known to regulate genes associated with cleft palate including *Pvrl1*, *Fgfr2* and *Pdgfa* demonstrating for the first time that p63 transcriptionally regulates these genes in the palatal epithelia. The observation that several genes required for periderm development are direct targets of p63 led us to hypothesize that p63 signalling is a master regulator of periderm formation and/or maintenance in the secondary palate. *p63*^-/-^ mice lack flattened periderm cells indicating that p63 signalling is critical for the ontogeny of this transient epithelial layer and our results demonstrate that p63 functions in two phases: p63 is initially required for periderm formation via regulation of *Irf6*, *Ripk4*, *Sfn*, *Grhl3* and *Jag2* and subsequently functions to maintain adhesion between the periderm and basal epithelia *via* regulation of a variety of cell adhesion molecules including the genes encoding desmosomal components such as plakoglobin, plakophilin-1 and plakophilin-3, and adherens junction components such as cadherin-3 and nectin-1.

Paradoxically, previous reports have demonstrated that failure of periderm formation and/or maintenance leads to pathological adhesions between apposed epithelial surfaces, a phenotype that is not observed in *p63*^-/-^ mice [8,14,31]. Given the critical role that p63 plays in palatal adhesion, we postulate that down-regulation of a large number of adherens junction- and desmosome-associated genes in the basal epithelia compromises epithelial adhesion in *p63*^-/-^ mice thereby preventing intraoral fusions from occurring despite the absence of periderm. In contrast, in *Irf6* mutant mice, where periderm is absent, adhesion protein complex proteins are up-regulated and mis-localized along the exposed apical epithelial surfaces leading to pathological adhesions [5,7,8].

Given the role of periderm is to prevent pathological adhesions between intimately apposed, adhesion-competent epithelia during development [5,8,43], this cell layer must be removed from the MEE to permit fusion of the palatal shelves. Previous results have suggested that the periderm cells covering the MEE either slough away from the basal cells prior to contact of the horizontally-positioned palatal shelves [44] or migrate out of the MES to the oral and nasal epithelial triangles [11]. Here, we have used a combination of immunohistochemistry and *ex vivo* imaging to demonstrate that initial contact between the horizontal palatal shelves is achieved via the periderm cells which subsequently migrate out of the MES. Moreover, the latter process does not occur in *Tgfb3*^-/-^ embryos in which the palatal shelves remain cleft. Intriguingly, while p63 is down-regulated in the MEE of wild-type embryos, p63 expression is maintained in the *Tgfb3*^-/-^ MEE and the periderm cells fail to migrate to the oral and nasal epithelial triangles [12, 19] (Figs 1 and 2).

Previously, it has been shown that the polarized expression of the adherens junction protein nectin-1 between the basal cells and periderm is lost prior to palatal shelf fusion in wild-type mice but these processes do not occur in the presence of the TGFβ inhibitor SB431542 [45]. Based on these observations, together with the data presented here, we hypothesize that p63 signalling promotes adherens junction and desmosome formation to maintain periderm/basal cell adhesion during the initial stages of palatal development. Subsequent TGFβ3-induced down-regulation of p63 after palatal shelf elevation leads to loss of adhesion between the periderm and basal cells, enabling periderm cells to migrate from the MEE into the nasal and oral epithelial triangles, thus ensuring proper fusion of the palatal shelves. In support of this hypothesis, reducing the level of p63 in the MEE of *Tgfb3*^-/-^ embryos restored the migratory periderm phenotype and rescued the cleft palate phenotype in *Tgfb3*^-/-^;*p63*^+/-^ mice. Notably, rescue of the cleft palate phenotype in *Tgfb3*^-/-^;*p63*^+/-^ mice also restored *Mmp13* expression to the MEE. Although other groups have demonstrated that *Mmp13* is transcriptionally regulated by p63 in SaOs-2 Tet-on cells [46], we were unable to identify any p63 binding sites near to the *Mmp13* promoter in our palatal ChIP-seq dataset or in data from primary [41], proliferating or differentiated keratinocytes [37]. These observations suggest that although *Mmp13* lies downstream of ΔNp63α, it is either controlled by a long-distance enhancer or it may not be a direct transcriptional target, at least in the palatal epithelia.

To provide further support for these observations we expressed ΔNp63α ectopically in the palatal epithelia *in vivo* and demonstrated that the resulting embryos exhibited sub-mucous cleft palate in which the MEE failed to degenerate. Instead, the palatal shelves were composed of a slightly expanded p63-positive basal layer covered by a thick layer of persistent periderm cells (Fig 6). Despite the fact that both *Tgfb3*^-/-^ and *Krt5*-tTA;pTRE-ΔNp63α embryos display persistent periderm and p63 expression, there are phenotypic differences between the two models, suggesting that TGFβ3 has p63-independent roles in regulating the fate of palatal epithelial cells.

To provide insights into the p63-mediated events underlying MEE degeneration, we intersected the results of microarray analysis of the palatal shelves of *Krt5*-tTA;pTRE-ΔNp63α embryos with the ChIP-seq analysis and identified a number of p63 transcriptional targets, including *Jag2*, *Znf750*, *Perp* and *Bcl11b*, which were upregulated in the MEE of the bi-transgenic embryos. *Jag2*, which is important for periderm formation, is down-regulated in the MEE at E14.5 with expression being maintained in the MEE of *Tgfb3*^-/-^ palatal shelves [43,47]. As we have now demonstrated that *Jag2* is a direct transcriptional target of p63 in the palatal epithelia and that *Jag2* expression is maintained in the MEE of *Krt5*-tTA;pTRE-ΔNp63α mice which fail to degenerate (Fig 7), we propose a model in which failure of TGFβ3-induced down-regulation of p63 leads to loss of *Jag2-Notch1*-induced periderm migration from the MES which contributes to the sub-mucous cleft palate observed in ΔNp63α over-expressing mice. In this context, we have also shown that *Znf750* expression, a known p63 transcriptional target in keratinocytes [48], is maintained in the MEE of *Krt5*-tTA;pTRE-ΔNp63α mice. Previous research has shown that ZNF750 induces KLF4, appropriate function of which is important in periderm differentiation [49].

In addition to its role in periderm development, p63 plays a major role in maintaining the proliferative potential of palatal epithelial cells in part by transcriptional regulation of the cell cycle regulator *Cdkn1a* [50]. However, analysis of the ChIP-seq data shows that p63 binds to an active enhancer ~48 kb upstream of *Bcl11b* which encodes the COUP-TF interacting protein CTIP2. Loss of Ctip2 results in reduced proliferation and increased apoptosis in mouse keratinocytes [51]. Notably, expression of Ctip2, which is normally down-regulated in the MEE of wild-type mice coincident with loss of p63 expression, is maintained when ΔNp63α is over-expressed. These observations support the concept that transcriptional regulation of *Bcl11b* by p63 plays a central role in the fate of the palatal epithelia.

In summary, together with published data, our observations support a model whereby ΔNp63α controls the spatio-temporal regulation of palatal epithelial cell fate to ensure appropriate palatal adhesion: p63-induced periderm formation and subsequent maintenance prevents premature adhesion between immature, adhesion-competent intra-oral epithelia, TGFβ3-induced down-regulation of p63 in the MEE driving Jag2-induced periderm migration to the oral and nasal epithelial triangles thereby preventing sub-mucous cleft palate. In addition, as observed in the epidermis, ΔNp63α plays a central role in maintaining the proliferative potential of the basal layer of the MEE, down-regulation of p63 leading to cell cycle arrest and cell death.

## Methods

### Maintenance and housing of mutant mice

All mice were housed with free access to food (Nutrients Rat and Mouse Standard Diet no.1 expanded; Ban Kingman) and water at the University of Manchester at a temperature of 20–22°C, with a humidity of 40–60% under specified mouse pathogen free conditions with 12 hours light/dark cycle. Genotyping of the BALB/c *p63* mouse line has been described previously [14].*Tgfb3* mutant mice have been described previously [23]; genotyping was achieved using modified primers (S5 Table) and the mice were maintained on a mixed CD1/C57Bl/6J background. *mKrt17*-GFP, *Krt5*-tTA and pTRE-ΔNp63α mice, maintained on a C57Bl/6J background, have been described previously [26,34–36]. Embryos were dissected from time-mated pregnant female mice, the morning on which the vaginal plug was detected being designated embryonic day (E) 0.5. No randomisation was performed and all results were analysed by two independent researchers, one of whom was blind to the genotype of the samples.

### RNA sequencing

RNA was isolated from E13.5 and E14.5 mouse palatal shelves using the Qiagen RNeasy kit. RNA-Seq libraries were generated using the SOLiD^™^ Total RNA-Seq Kit. Samples were run on SOLiD^™^ v4 for single-end 50 bp reads. Poor reads were filtered from the data with SOLiD Preprocess Filter [52]. TopHat software [53] was used to align reads against the mm9 reference genome. The RNA-Seq data are available from ArrayExpress: E-MTAB-3157.

### ChIP-sequencing

Palatal shelves dissected from E13.5 and E14.5 mouse were cross-linked with 1% formaldehyde for 20 minutes. Chromatin was prepared by homogenizing the tissue in PBS using an Ultra-Turrax Homogenizer and incubating the resultant cells in PIPES buffer according to the Upstate ChIP assay protocol (Merck Millipore). Chromatin was sonicated using a Sonics Vibracell with seven 10 second pulses set to an amplitude of 40. Chromatin was prewashed with protein A-agarose beads (SC-2001; Santa Cruz,) and incubated with 2 μg of α isoform-specific p63 antibody (H129; Santa Cruz) overnight at 4°C. Antibody-bound chromatin was recovered using protein A-agarose beads (Santa Cruz). Sample preparation for sequencing was performed according to the manufacturer’s instructions (Illumina) and sequenced using the Illumina GAII. 32 base-pair single end reads were mapped to the mouse genome (mm9, NCBI Build 37) using bowtie 0.12.7 [54] reporting unique, full-length alignments with a maximum of 1 nucleotide mismatch to the reference sequence. Mapped reads were peak-called using MACS 1.3.7 [55] with default parameters and a *P* value threshold of 1 x 10^−5^. Binding sites were associated to genes using RnaChipIntegrator (Briggs PJ, Donaldson IJ, Zeef LAH. RnaChipIntegrator, available at: https://github.com/fls-bioinformatics-core/RnaChipIntegratorversion0.3.2).

### Microarray analysis

Amplified sense-strand cDNA was generated from 100 ng of total RNA (Ambion WT Expression Kit). Fragmentation, labelling (Affymetrix Genechip WT Terminal labelling kit), and subsequent hybridization utilizing Affymetrix Genechip Mouse Exon 1.0 ST Array was performed in the Genomic Technologies Core Facility, University of Manchester. Microarray data were analyzed using Partek Genomics Solution (version 6.6, Partek Inc. St. Charles, MO, USA). Probe sets of a core subset were quantile normalized and Robust Multi-array Average (RMA) background correction applied. Exons were summarized to genes by calculating the mean of the exons (log 2). To establish relationships and compare variability between replicate arrays and experimental conditions, principal components analysis (PCA) was used [56]. Correction for false discovery rates was completed using the method of QVALUE [57]. Heatmaps were generated for 62 genes. These genes were clustered based on expression profiles. For each sample, expression levels (log2) were normalized prior to clustering by z-transformation (mean = 0, Standard deviation = 1 for each gene). Genes were segregated into two clusters using a K-means algorithm (Manhattan distance) followed by hierarchical clustering of each resulting K-means cluster using the maxdView software ‘Super Grouper’ plugin (available from http://bioinf.man.ac.uk/microarray/maxd/).

### qPCR analysis

RNA was extracted as above and total RNA transcribed into cDNA according to the manufacturer’s protocol (Invitrogen). Primers were designed using Primer 3 (http://frodo.wi.mit.edu), and qPCR reactions were performed using Power SYBR Green PCR Master Mix according to the manufacturer’s protocol (Life Technologies). For qPCR analysis of cDNA, exon-spanning primer sets for *p63* candidate target genes were used (S6 Table). Mouse β-actin was used to normalize the amount of cDNA. Differences in relative transcript expression between wild-type and *p63* mutant samples were calculated by the 2^ΔΔCt^ method [58]. Statistical significance was assessed using the Mann Whitney U test with at least five biological replicates for each genotype.

For qPCR of ChIP results, one primer set was used for each tested binding region (S6 Table). Each binding region was tested using chromatin extracted from E14.5 palatal shelves using at least three independent litters to ensure appropriate biological replicates. ChIP efficiency of binding sites was calculated using percentage of immunoprecipitated DNA against input chromatin. Occupancy used in ChIP experiments was calculated using ChIP efficiency of candidate binding regions standardized against that of a region within exon 2 of the myoglobin gene. Statistical significance was assessed using the Students T-test.

### Histological analysis, immunofluorescence and in situ hybridization

Heads dissected from E12.5, E13.5 and E14.5 mice were fixed in 4% paraformaldehyde and processed for histological examination using standard protocols. For immunofluorescence analyses, sections were treated with 10 mm citrate buffer at 96°C for 10 min for antigen retrieval. Sections were incubated overnight at 4°C with antibodies against E-cadherin (1:200; Clone-36, BD Bioscience), keratin 17 (1/1000), keratin 14 (1/200, clone MS-115-P1, Neomarkers) nectin-1, (1:200; Santa Cruz SC-28639), nectin-4 (1:100; HPA010775 Sigma), plakoglobin (1/200; clone 15F11 Sigma), p63 (1:1000; Santa Cruz 4A4) and Bcl11b (1/500 Ctip2, clone 25B6, Abcam). *In situ* hybridization was performed as described previously with detection using BM Purple (Roche) [8]. Sections were counterstained and visualized using a Leica DMRB microscope. Littermates were used as controls wherever possible, in order to correctly stage the mutant (*Tgfb3*^-/-^;*p63*^+/-^ or *Krt5*-tTA;pTRE-ΔNp63α bi-transgenic embryos), and to provide control tissue. In all cases, the histological analyses were performed on at least five mutant embryos from all gestational ages (two for the neonatal *Krt5*-tTA;pTRE-ΔNp63α bi-transgenic embryos due to ethical considerations). Immunofluorescence and in situ hybridization assays were performed in duplicate.

### Time-lapse confocal imaging

Palatal shelves were dissected from E14.0 embryos and cultured on filters suspended on serum-free DMEM/F-12 medium. Palatal shelves were allowed to contact for 3 hours at 37°C in 5% CO_2_ before being embedded in 5% UltraPure Low Melting Point Agarose (Life Technologies) in DMEM/F-12 medium. Whole palates were then excised from the agarose gel in blocks and sliced at the midpoint of the antero-posterior axis of the palatal shelves. Slices were positioned mid-palate down in a 35 mm petri dish (Thermo Scientific) and covered in 1% UltraPure Low Melting Point Agarose in DMEM/F-12 medium. Images were collected over a 24 hour period on a Nikon C1 confocal using a TE2000 PSF inverted microscope and a 10x/0.50 Plan Fluor objective. The confocal settings were as follows, pinholes 30 μm, scan speed 400 Hz unidirectional, format 1024 x 1024. Images for FITC were excited with the 488 nm laser line. When acquiring 3D optical stacks, the confocal software was used to determine the optimal number of Z sections. Only the maximum intensity projections of these 3D stacks are shown in the results. Images were acquired on a Cascade 512 EM CCD camera (Photometrics) through the Elements Software (Nikon).

## Supporting information

S1 Fig Palatal development in E13.5 wild-type, *Tgfb3*^-/-^ and *Tgfb3*^-/-^;*p63*^+/-^ mice. (**A**—**C**) The palatal shelves of wild-type, *Tgfb3*^-/-^ and *Tgfb3*^-/-^;*p63*^+/-^ mice lie in a vertical position lateral to the tongue. (**D**—**I**) While the basal epithelial cells are proliferative and express E-cadherin and p63, there is no evidence of cell death. (**J**—**L**) In all genotypes, the palatal epithelia consist of a keratin 14-positive basal layer covered by a distinct keratin 17-positive layer of periderm cells. p: palatal shelves; t: tongue. Scale bars: A-C, 250 μm; D-L, 100 μm.
(TIF)

S2 Fig Histological analysis of E15.0 wild-type, *Tgfb3*^-/-^ and *Tgfb3*^-/-^;*p63*^+/-^ mice. Representative images taken from serial sections of wild-type, *Tgfb3*^-/-^ and *Tgfb3*^-/-^;*p63*^+/-^ embryos in the anterior (**A-C**), mid (**D-F**) and posterior (**G-I**) regions of the palate at E15.0. p: palatal shelves; t: tongue. Scale bars: 250 μm.
(TIF)

S3 Fig Percentage of proliferative cells in the palatal shelves of wild-type and *Tgfb3*^-/-^;*p63*^+/-^ embryos at E14.5. Proliferative cells were assessed by phosphohistone 3 immunostaining and the percentages calculated for the epithelium, mesenchyme and total palate. No significant differences were found between the wild-type littermate controls and the *Tgfb3*^-/-^;*p63*^+/-^ embryos: epithelium, P = 0.21; mesenchyme P = 0.37; total palate P = 0.98, all Student’s T Test, n = 3.
(TIF)

S4 Fig Genome-wide distribution of ΔNp63α binding sites in the secondary palate. (**A**) RNA-seq analysis indicates that transcripts encoding ΔNp63 isoforms predominate in the developing secondary palate. Transcript reads are indicated by black bars. (**B**) ChIP-qPCR validation of p63-bound sites. Fold-enrichment for each binding region was calculated relative to a control region in exon 2 of myoglobin (set at 1; pale bar), to which p63 does not bind. Asterisks represent the level of significance: * = P <0.05, ** = P <0.01, *** = P <0.001; Student’s t-test, n = 4. (**C**) p63 binding site distribution relative to RefSeq genes. Binding site regions are divided into TSS flanking region (5 kb upstream of TSS, first exon & first intron), intragenic region (all introns and exons excluding first), <25 kb (5–25 kb upstream or 25 kb downstream of last exon), or intergenic regions. (**D**) GREAT functional annotation of the genes associated with the p63-bound regions.
(TIF)

S5 Fig Development of the palatal shelves in E13.5 *Krt5*-tTA;pTRE-ΔNp63α bi-transgenic mice. (**A**, **B**) The palatal shelves of wild-type and *Krt5*-tTA;pTRE-ΔNp63α embryos lie in a vertical position lateral to the tongue. (**C**, **D**) In both genotypes, the palatal epithelia consist of a keratin 14-positive basal layer covered by a distinct keratin 17-positive layer of periderm cells. (**E**—**J**) The palatal epithelia are proliferative and express E-cadherin and p63. p: palatal shelves. Scale bars: A-C, 250 μm; D-L, 100 μm.
(TIF)

S6 Fig Percentage of proliferative cells in the palatal shelves of wild-type and *Krt5*-tTA;pTRE-ΔNp63α bi-transgenic mice at E13.5. Proliferative cells were assessed by phosphohistone 3 immunostaining and the percentages calculated for the epithelium, mesenchyme and total palate. No significant differences were found between the wild-type littermate controls and the *Tgfb3*^-/-^;*p63*^+/-^ embryos: epithelium, P = 0.97; mesenchyme P = 0.26; total palate P = 0.31, all Student’s T Test, n = 3.
(TIF)

S7 Fig Histological analysis of E15.0 wild-type and *Krt5*-tTA;pTRE-ΔNp63α bi-transgenic mice. Representative images taken from serial sections of wild-type and *Krt5*-tTA;pTRE-ΔNp63α bi-transgenic embryos in the anterior (**A-C**), mid (**D-F**) and posterior (**G-I**) regions of the palate at E15.0. C, F and I are magnified regions of the boxes represented in B, E and H. p: palatal shelves; t: tongue. Scale bars: A, B, D, E, G, H = 250 μm and C, F and I = 100 μm.
(TIF)

S8 Fig *Krt5*-tTA;pTRE-ΔNp63α bi-transgenic mice exhibit sub-mucous cleft palate. (**A**) In neonatal wild-type mice, the medial edge epithelia have degenerated to allow mesenchymal continuity across the secondary palate. (**B**) In contrast, in 50% of neonatal *Krt5*-tTA;pTRE-ΔNp63α mice, the medial edge epithelia remain intact leading to sub-mucous cleft palate (arrowed). (**C, D**) Immunostaining with anti-HA and anti-ΔNp63↑ antibodies confirms that the transgene is expressed ectopically in the medial edge epithelia of E15.5 Krt5-tTA;pTRE-ΔNp63α mice (arrowed) thereby restoring ΔNp63↑ expression in these cells. p: palatal shelves. Scale bars: 100 μm.
(TIF)

S9 Fig Percentage of proliferative cells in the palatal shelves of E14.5wild-type and *Krt5*-tTA;pTRE-ΔNp63α embryos. Proliferative cells were assessed by anti-BrdU immunostaining and the percentages calculated for the epithelium, mesenchyme and total palate. No significant differences were found between the wild-type controls and the *Tgfb3*^-/-^;*p63*^+/-^ embryos: epithelium, P = 0.31; mesenchyme P = 0.42; total palate P = 0.29, all Student’s T Test, n = 3.
(TIF)

S1 Video Time-lapse imaging of palatal shelves dissected from *mKrt17*-GFP transgenic mice. The periderm exhibits a migratory phenotype allowing completion of palatal fusion in wild-type mice. The video is a series of representative images taken at the same Z position over a 24-hour culture period.
(MP4)

S2 Video Time-lapse imaging of palatal shelves dissected from *mKrt17*-GFP;*Tgfb3*^-/-^ embryos in which the secondary palate remains cleft. No migration of the periderm cells was observed. The video is a series of representative images taken at the same Z position over a 24-hour culture period.
(MP4)

S3 Video Time-lapse imaging of palatal shelves dissected from *mKrt17*-GFP;*Tgfb3*^-/-^;*p63*^+/-^embryos. Reducing p63 dosage in *Tgfb3*^-/-^ mice restores periderm migration and palatal fusion thereby rescuing the cleft palate phenotype. The video is a series of representative images taken at the same Z position over a 24-hour culture period.
(MP4)

S1 Table Classification of the 6295 genomic regions bound by ΔNp63α in the palatal shelves of wild-type embryos. (XLSX)

S2 Table Results of the microarray analysis of palatal shelves dissected from E14 wild-type *versus p63*^-/-^ mice. (XLS)

S3 Table Genes encoding proteins implicated in cell adhesion that are differentially-expressed in wild-type *versus p63*^-/-^ mice. (XLSX)

S4 Table Results of the microarray analysis of palatal shelves dissected from E14.5 wild-type *versus Krt5*-tTA;pTRE-ΔNp63α mice. (XLSX)

S5 Table A list of 104 genes that exhibit diametric expression with down-regulation in the p63 loss-of-function (*p63*^-/-^) microarray analysis and up-regulation in the *Krt5*-tTA;pTRE-ΔNp63α microarray analysis. (XLSX)

S6 Table Sequences of the oligonucleotide primers used in the study. (XLSX)
