# Supplementary figures and images for: p63 exerts spatio-temporal control of palatal epithelial cell fate to prevent cleft palate
Source: PLoS Genet. 2017 Jun 12;13(6):e1006828. doi: 10.1371/journal.pgen.1006828 (PMC5484519; doi:10.1371/journal.pgen.1006828)

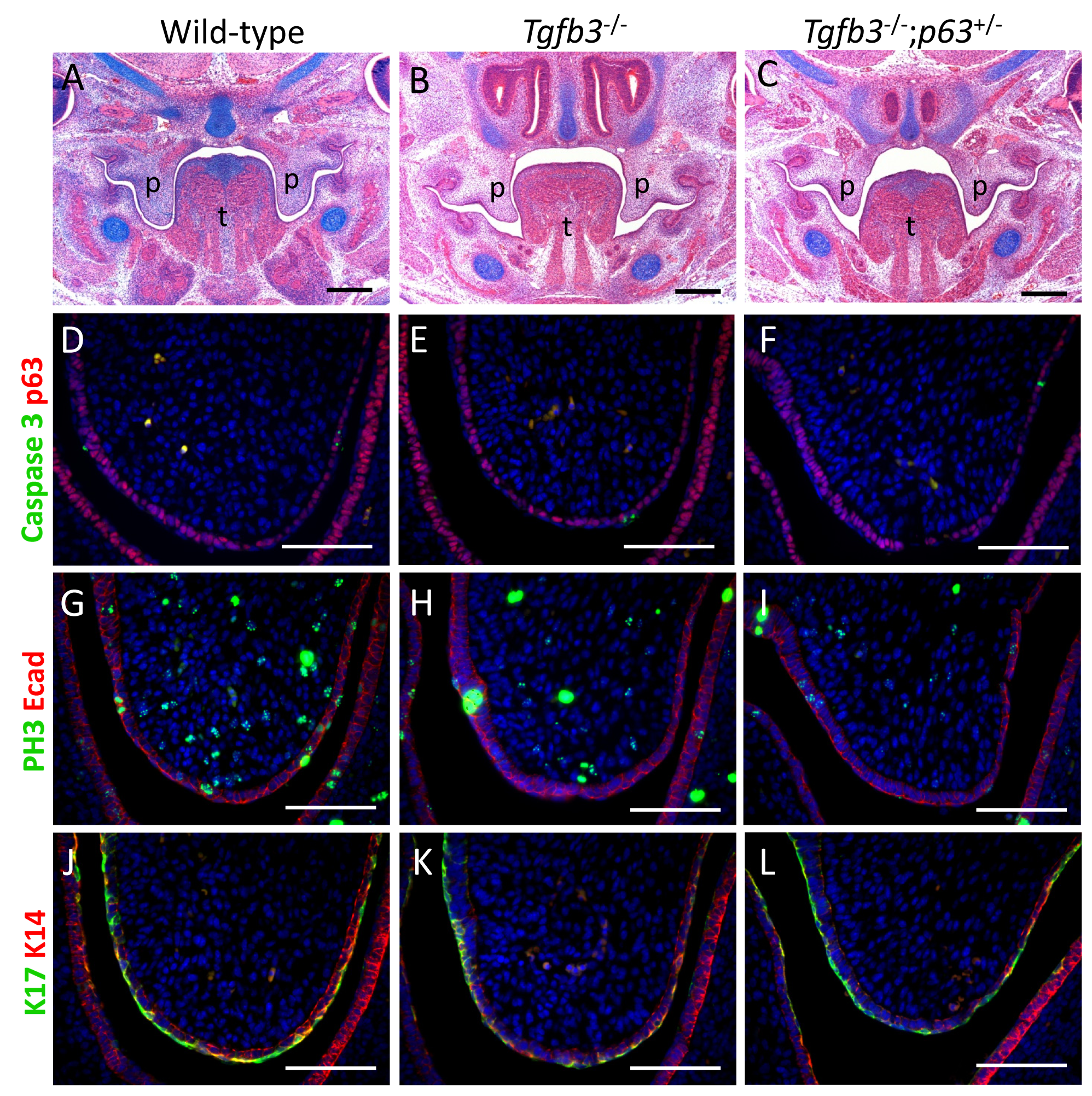

Supplement: S1 Fig — (A—C) The palatal shelves of wild-type, Tgfb3-/- and Tgfb3-/-;p63+/- mice lie in a vertical position lateral to the tongue. (D—I) While the basal epithelial cells are proliferative and express E-cadherin and p63, there is no evidence of cell death. (J—L) In all genotypes, the palatal epithelia consist of a keratin 14-positive basal layer covered by a distinct keratin 17-positive layer of periderm cells. p: palatal shelves; t: tongue. Scale bars: A-C, 250 μm; D-L, 100 μm. (TIF) [file pgen.1006828.s001.tif]

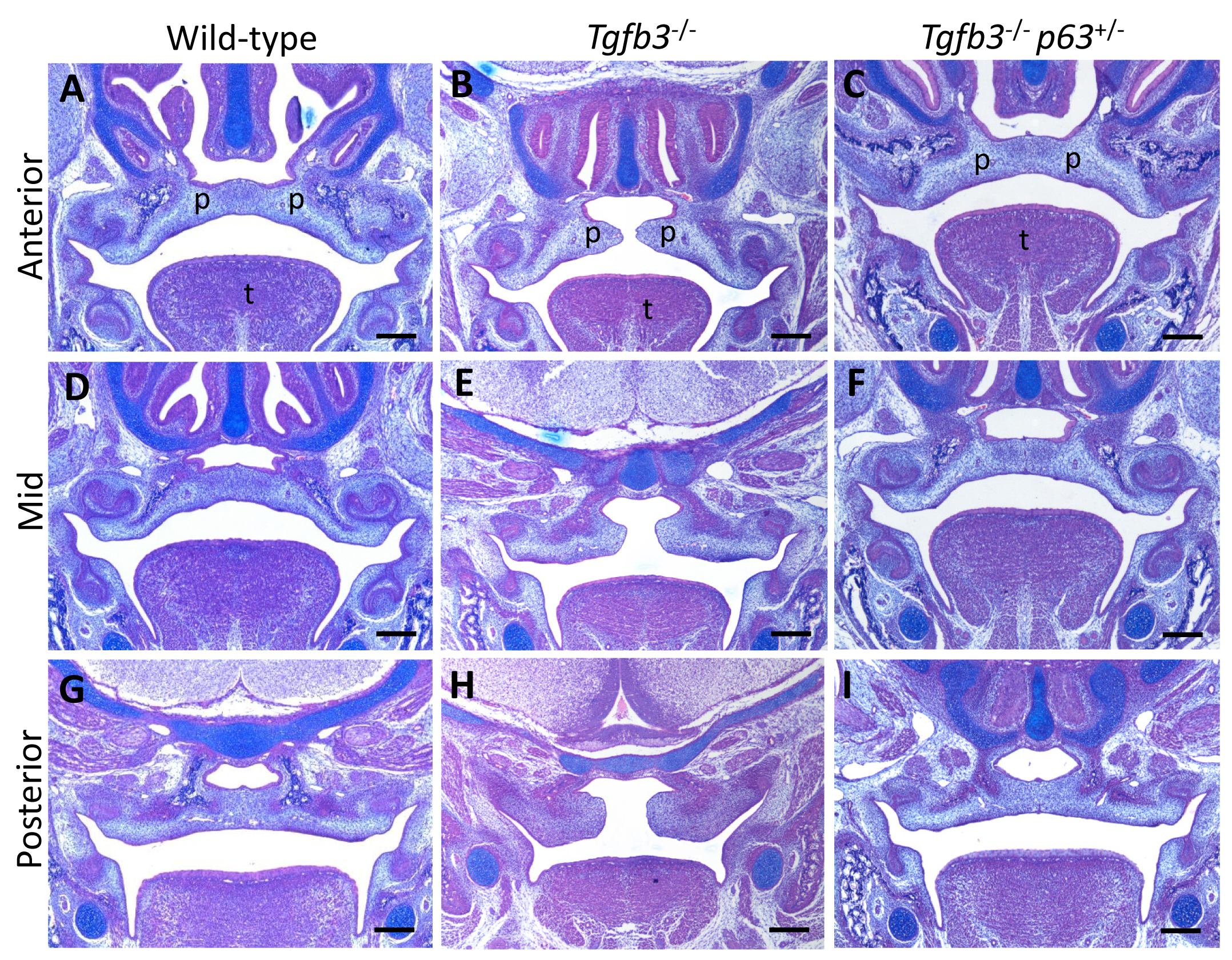

Supplement: S2 Fig — Representative images taken from serial sections of wild-type, Tgfb3-/- and Tgfb3-/-;p63+/- embryos in the anterior (A-C), mid (D-F) and posterior (G-I) regions of the palate at E15.0. p: palatal shelves; t: tongue. Scale bars: 250 μm. (TIF) [file pgen.1006828.s002.tif]

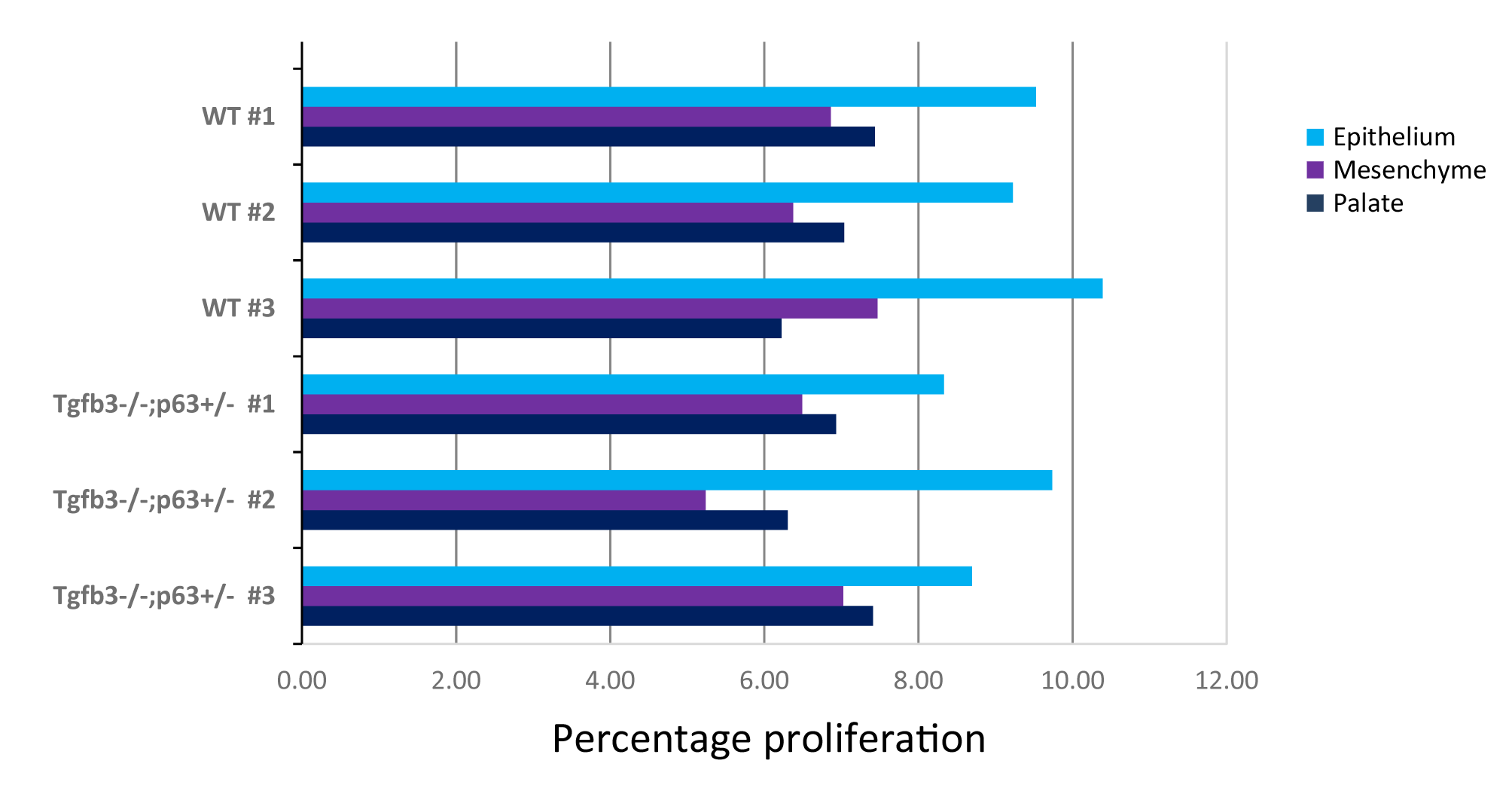

Supplement: S3 Fig — Proliferative cells were assessed by phosphohistone 3 immunostaining and the percentages calculated for the epithelium, mesenchyme and total palate. No significant differences were found between the wild-type littermate controls and the Tgfb3-/-;p63+/- embryos: epithelium, P = 0.21; mesenchyme P = 0.37; total palate P = 0.98, all Student’s T Test, n = 3. (TIF) [file pgen.1006828.s003.tif]

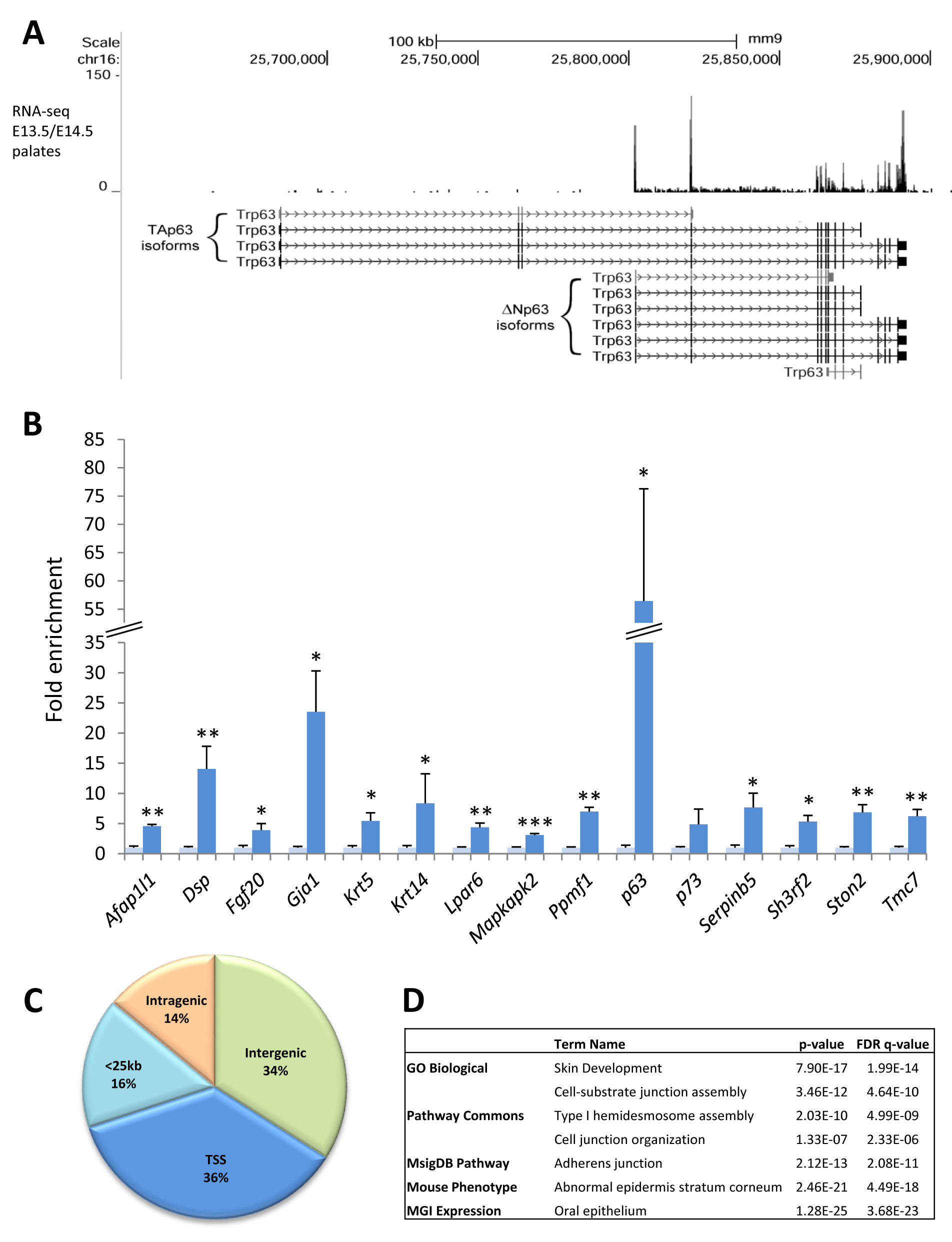

Supplement: S4 Fig — (A) RNA-seq analysis indicates that transcripts encoding ΔNp63 isoforms predominate in the developing secondary palate. Transcript reads are indicated by black bars. (B) ChIP-qPCR validation of p63-bound sites. Fold-enrichment for each binding region was calculated relative to a control region in exon 2 of myoglobin (set at 1; pale bar), to which p63 does not bind. Asterisks represent the level of significance: * = P <0.05, ** = P <0.01, *** = P <0.001; Student’s t-test, n = 4. (C) p63 binding site distribution relative to RefSeq genes. Binding site regions are divided into TSS flanking region (5 kb upstream of TSS, first exon & first intron), intragenic region (all introns and exons excluding first), <25 kb (5–25 kb upstream or 25 kb downstream of last exon), or intergenic regions. (D) GREAT functional annotation of the genes associated with the p63-bound regions. (TIF) [file pgen.1006828.s004.tif]

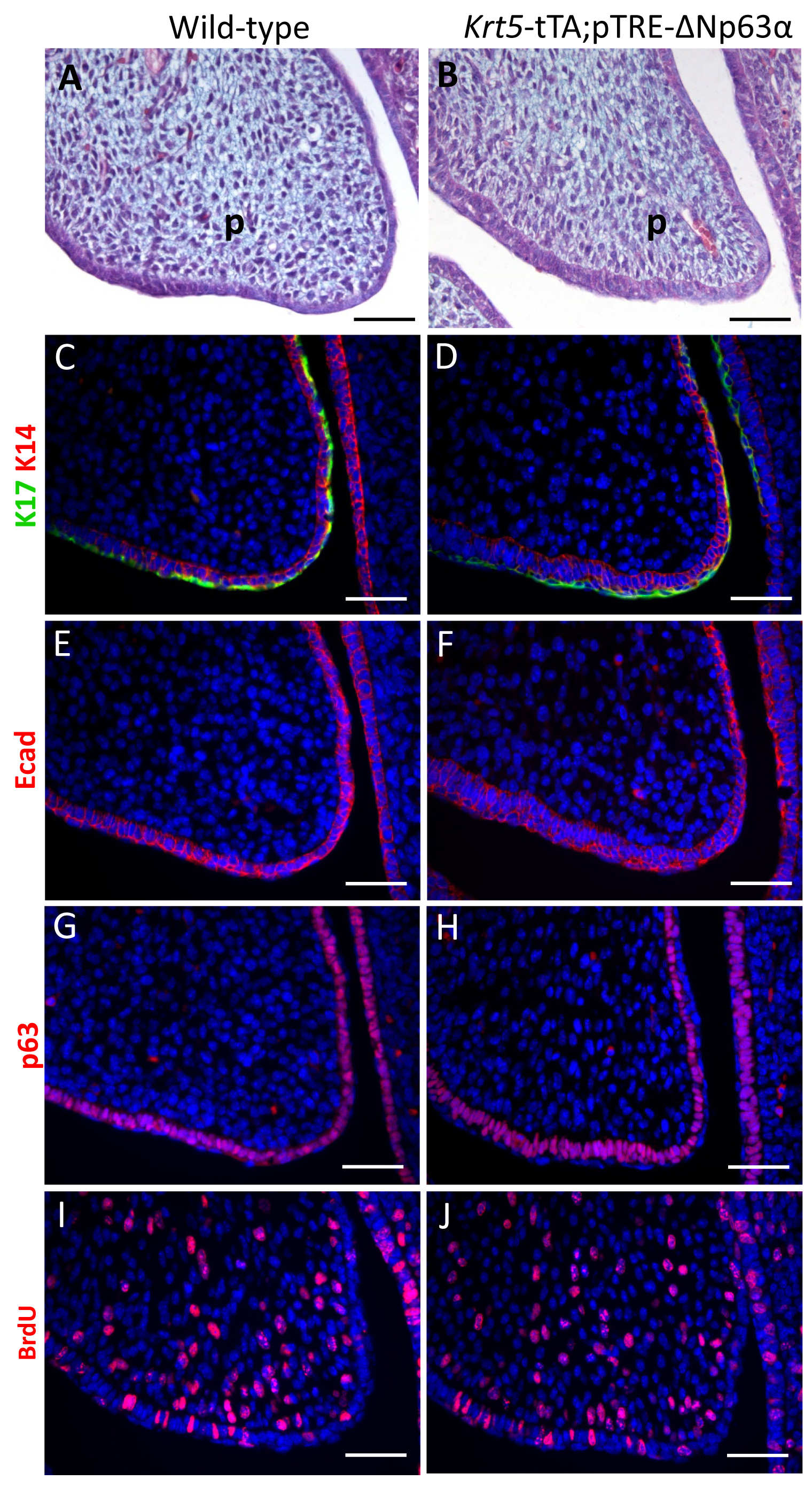

Supplement: S5 Fig — (A, B) The palatal shelves of wild-type and Krt5-tTA;pTRE-ΔNp63α embryos lie in a vertical position lateral to the tongue. (C, D) In both genotypes, the palatal epithelia consist of a keratin 14-positive basal layer covered by a distinct keratin 17-positive layer of periderm cells. (E—J) The palatal epithelia are proliferative and express E-cadherin and p63. p: palatal shelves. Scale bars: A-C, 250 μm; D-L, 100 μm. (TIF) [file pgen.1006828.s005.tif]

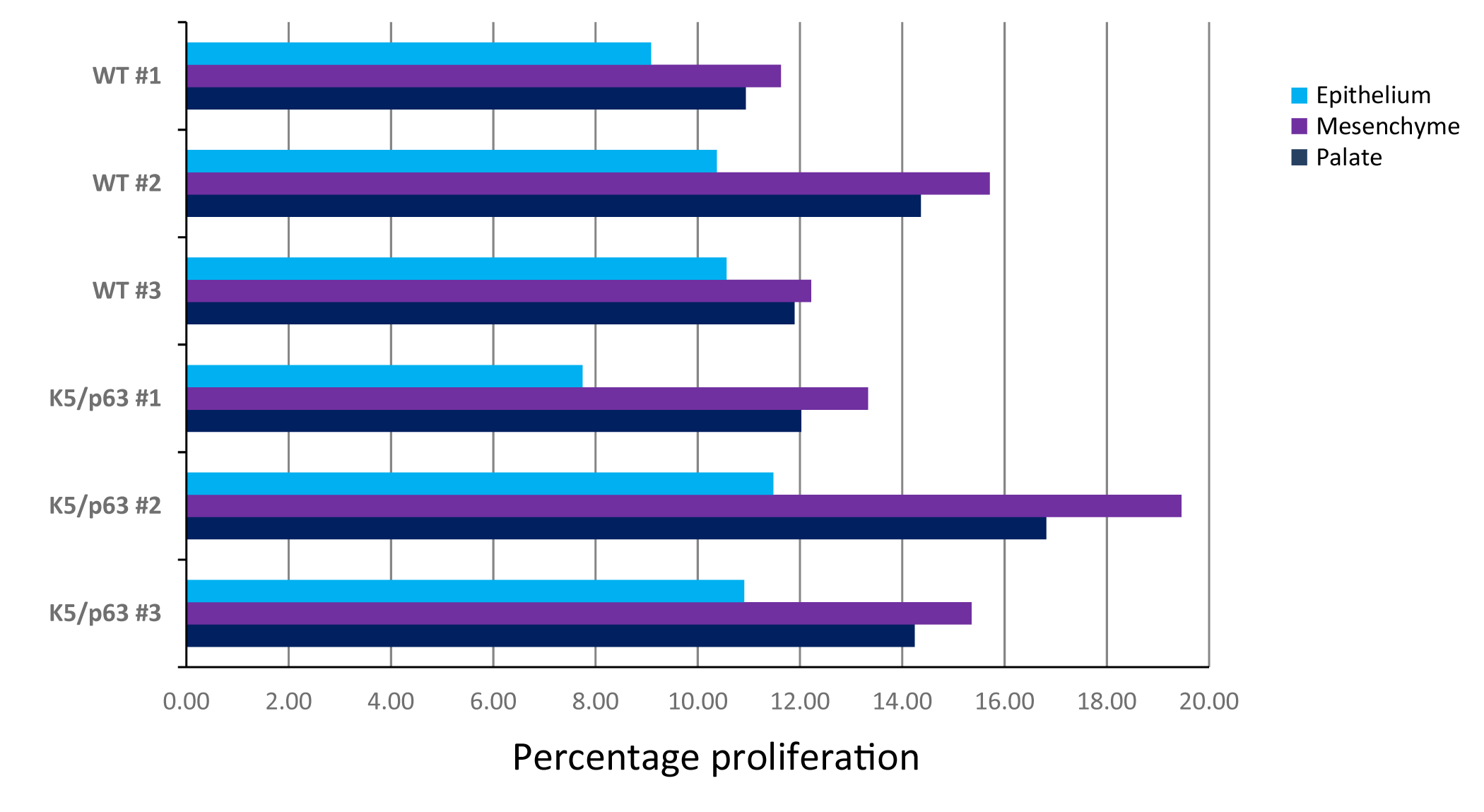

Supplement: S6 Fig — Proliferative cells were assessed by phosphohistone 3 immunostaining and the percentages calculated for the epithelium, mesenchyme and total palate. No significant differences were found between the wild-type littermate controls and the Tgfb3-/-;p63+/- embryos: epithelium, P = 0.97; mesenchyme P = 0.26; total palate P = 0.31, all Student’s T Test, n = 3. (TIF) [file pgen.1006828.s006.tif]

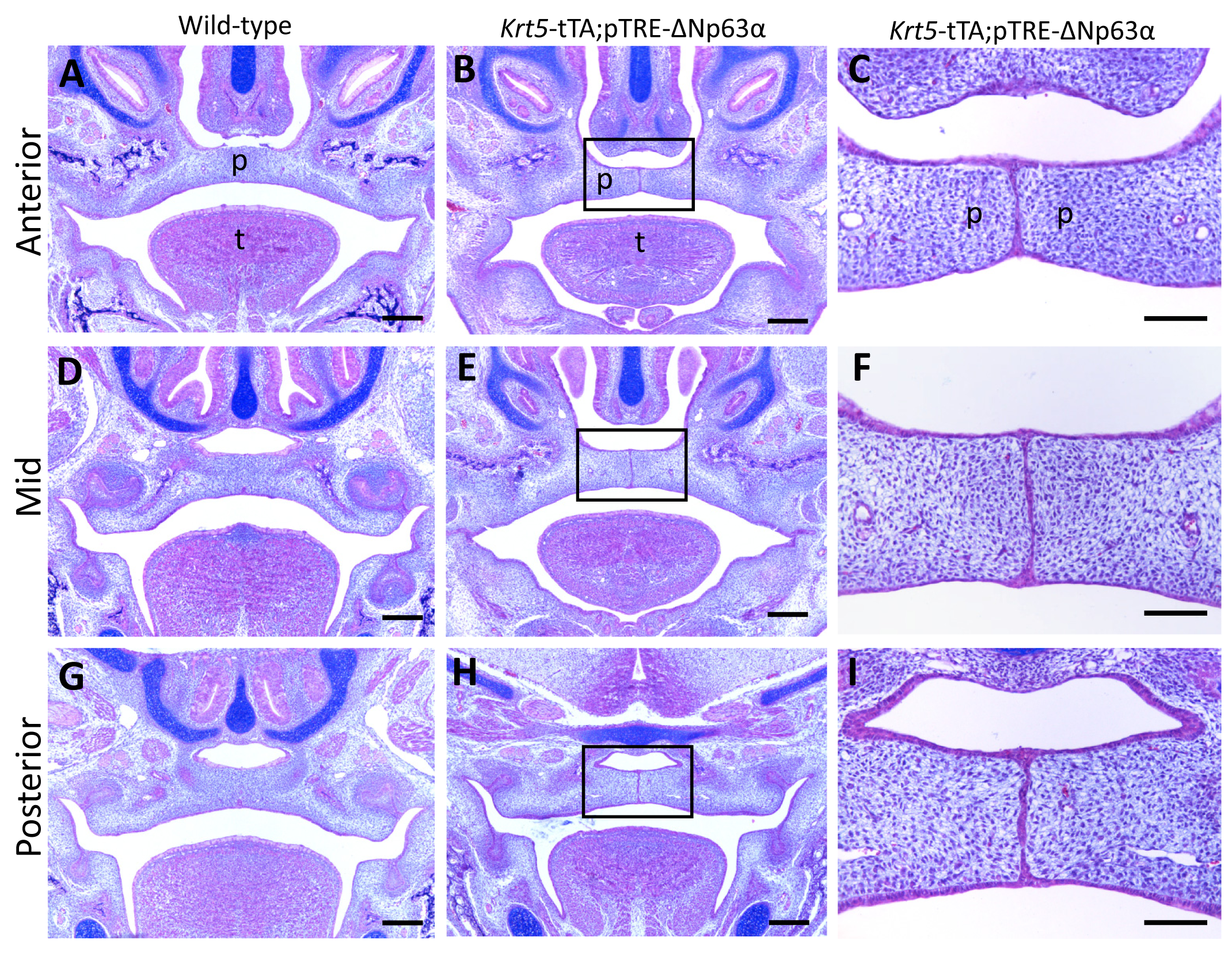

Supplement: S7 Fig — Representative images taken from serial sections of wild-type and Krt5-tTA;pTRE-ΔNp63α bi-transgenic embryos in the anterior (A-C), mid (D-F) and posterior (G-I) regions of the palate at E15.0. C, F and I are magnified regions of the boxes represented in B, E and H. p: palatal shelves; t: tongue. Scale bars: A, B, D, E, G, H = 250 μm and C, F and I = 100 μm. (TIF) [file pgen.1006828.s007.tif]

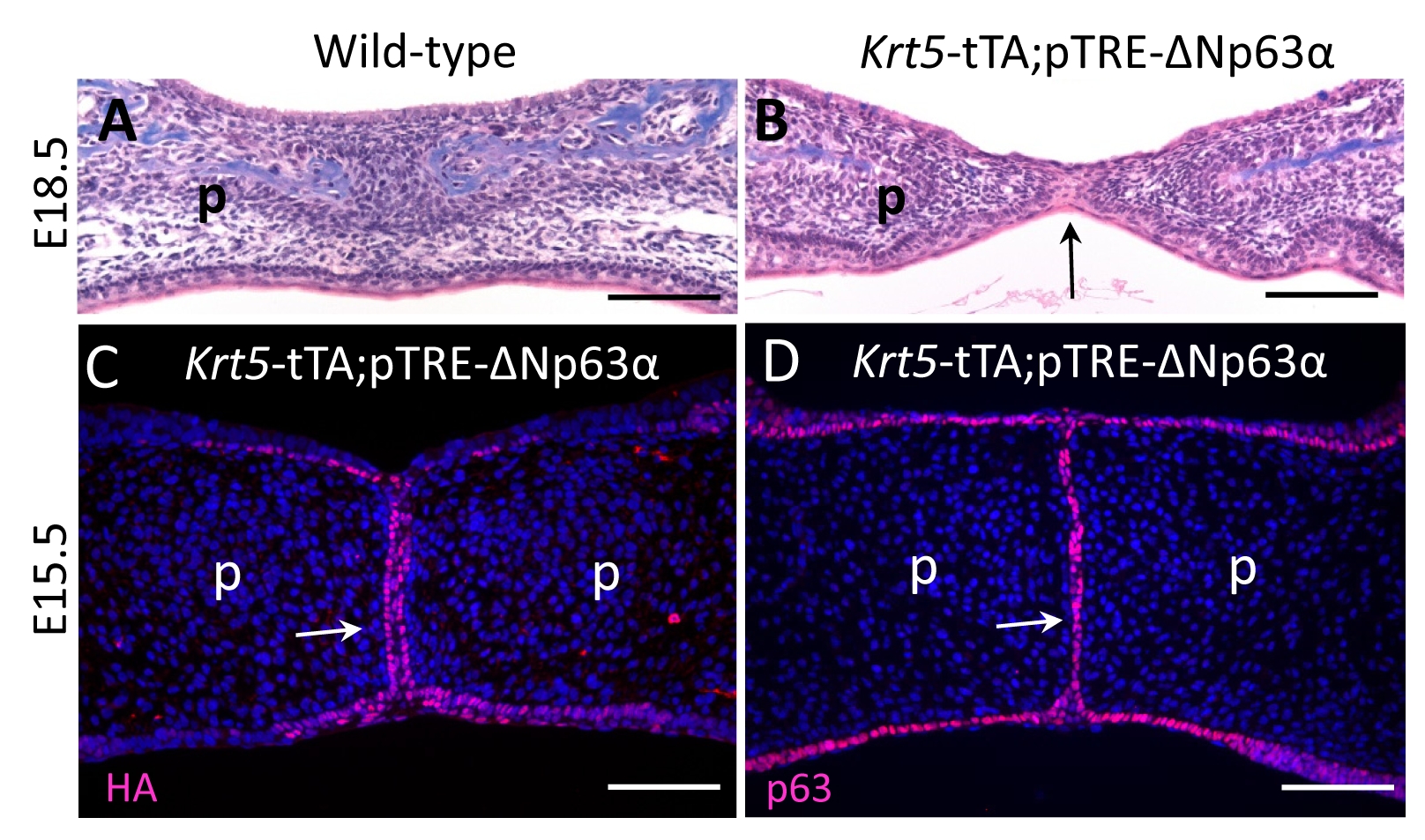

Supplement: S8 Fig — (A) In neonatal wild-type mice, the medial edge epithelia have degenerated to allow mesenchymal continuity across the secondary palate. (B) In contrast, in 50% of neonatal Krt5-tTA;pTRE-ΔNp63α mice, the medial edge epithelia remain intact leading to sub-mucous cleft palate (arrowed). (C, D) Immunostaining with anti-HA and anti-ΔNp63↑ antibodies confirms that the transgene is expressed ectopically in the medial edge epithelia of E15.5 Krt5-tTA;pTRE-ΔNp63α mice (arrowed) thereby restoring ΔNp63↑ expression in these cells. p: palatal shelves. Scale bars: 100 μm. (TIF) [file pgen.1006828.s008.tif]

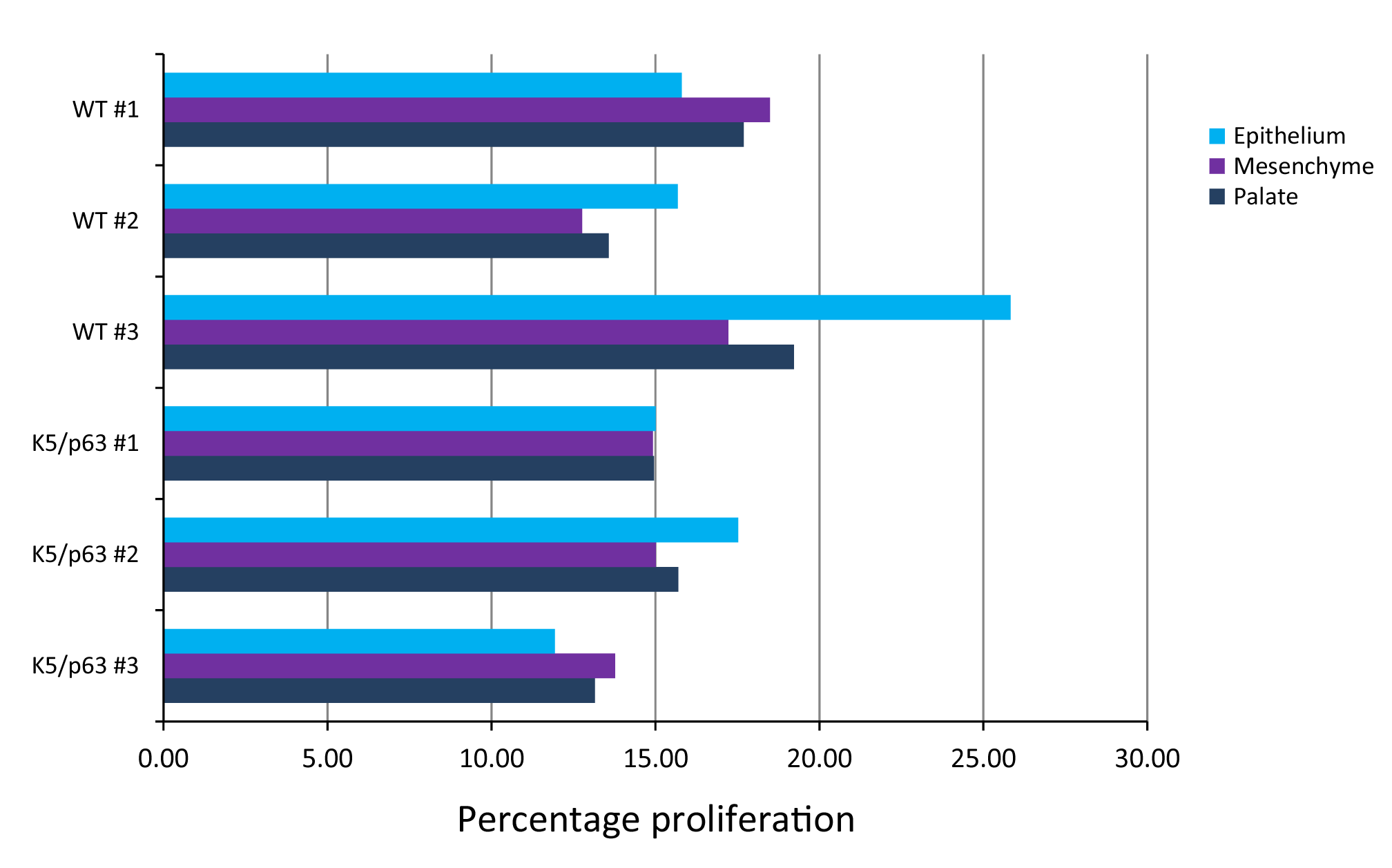

Supplement: S9 Fig — Proliferative cells were assessed by anti-BrdU immunostaining and the percentages calculated for the epithelium, mesenchyme and total palate. No significant differences were found between the wild-type controls and the Tgfb3-/-;p63+/- embryos: epithelium, P = 0.31; mesenchyme P = 0.42; total palate P = 0.29, all Student’s T Test, n = 3. (TIF) [file pgen.1006828.s009.tif]
